# Graph-Theoretic Analysis of Belief System Dynamics under Logic Constraints

**DOI:** 10.1038/s41598-019-45076-4

**Published:** 2019-06-20

**Authors:** Angelia Nedić, Alex Olshevsky, César A. Uribe

**Affiliations:** 1 School of Electrical, Computer and Energy Engineering, Arizona State University, Tempe, 85287 USA; 2 Department of Electrical and Computer Engineering, Division of Systems Engineering, and Center for Information and Systems Engineering, Boston University, Boston, 02215 USA; 3 Laboratory for Information and Decision Systems, and the Institute for Data Systems, and Society, Massachusetts Institute of Technology, Cambridge, 02139 USA

**Keywords:** Applied mathematics, Complex networks

## Abstract

Opinion formation cannot be modeled solely as an ideological deduction from a set of principles; rather, repeated social interactions and logic constraints among statements are consequential in the construct of belief systems. We address three basic questions in the analysis of social opinion dynamics: (i) Will a belief system converge? (ii) How long does it take to converge? (iii) Where does it converge? We provide graph-theoretic answers to these questions for a model of opinion dynamics of a belief system with logic constraints. Our results make plain the implicit dependence of the convergence properties of a belief system on the underlying social network and on the set of logic constraints that relate beliefs on different statements. Moreover, we provide an explicit analysis of a variety of commonly used large-scale network models.

## Introduction

The modeling of opinion dynamics spans several decades of interdisciplinary research^1–9^. Belief systems are typically modeled as a process where agents continuously update their opinions on a set of truth statements via repeated interactions, and opinions are exchanged following some social structure^10,11^. New opinions are formed by aggregating operations weighted by the relative importance assigned by an individual to others. This simple characterization has provided tools for analyzing the long-term behavior of belief systems using systems theory. Nevertheless, without significant modification, this framework has been shown insufficient to explain the existence of shared beliefs in a population^12^.

Recently proposed generalizations of opinion dynamics models integrate functional interdependencies among issues that coherently bound ideas and attitudes^13^. The existence of *logic constraints* in a belief system provides a successful model for the evolution of opinions in both large-scale populations and small groups^12^. *Logic constraints* build upon the natural idea that believing a specific statement is true may depend on the belief that some other related statements are true as well.

Understanding the role of the networks involved in the structural features of a belief system is of critical importance and can have direct implications for better decision-making and policy design^12,14–17^. Particularly, the fields on consensus dynamics and average consensus theory have provided a plethora of results analyzing the impact of the network structure with the opinion dynamic of a social network^18–22^. Even though sophisticated algebraic tools^13,21^ exists for the analysis of opinion dynamics, they can be unpractical or intractable for large-scale complex networks.

In this paper, we study how the structural properties of the social network of agents and the set of logic constraints influence the dynamics of a belief system from a *graph-theoretic* point of view. We describe the combinatorial features which influence the convergence of beliefs, the expected convergence time and the stationary value of the belief system. Informally, we answer the following three questions with graph-theoretic conditions that are easily accessible for a number of commonly used topologies in large-scale complex networks:

1. When does a belief system converge?
2. How long does it take for a belief system to converge?
3. Where does a belief system converge?

## Results

### Belief system with logic constraints

Friedkin *et al*.^12,13^ describe a belief system with logic constraints as a group of *n* agents that periodically exchange and update their opinions about a set of *m* different truth statements with logical dependencies among them. After each social interaction, the agents use shared opinions, as well as underlying logical dependencies among them, to update their beliefs.

The agents exchange their opinions by interacting over a social network captured by a graph ${\mathscr{G}}=(V,E)$, where *V* is the set of agents, and *E* is a set of edges. A directed edge towards an agent indicates that it receives the opinion of another agent, i.e., the directed flow of information. Analogously, the logical dependencies among the truth statements are modeled by a graph ${\mathscr{T}}=(W,D)$, where an edge between two statements exists if the belief in one statement affects belief in the other.

The generalized dynamics of a belief system are defined as follows. First, every agent aggregates its opinions on every truth statement according to the imposed logic constraints (i.e., modifying the opinions to consider the dependencies on the other truth statements). Second, the agents share their opinions over a social network, where the opinions are aggregated again to take into account those coming from the neighboring agents (i.e., social interactions). Finally, a new opinion is formed as a combination of the most recent aggregation and the initial opinion, modeling the adversity to deviate from the initial beliefs or stubbornness.

The aggregation steps consist of weighted (convex) combinations of the available values, where the weights represent the relative influence. This model is described in Eq. (1) for an arbitrary agent *I* ∈ *V* and an arbitrary statement *u* ∈ *W*:

1 $${\hat{x}}_{k}^{i}(u)=\sum _{v=1}^{m}\,{C}_{uv}{x}_{k}^{i}(v)\,({\rm{Aggregation}}\,{\rm{by}}\,{\rm{logic}}\,{\rm{constraints}})$$

2 $${\bar{x}}_{k}^{i}(u)=\sum _{j=1}^{n}\,{A}_{ij}{\hat{x}}_{k}^{j}(u)\,({\rm{Aggregation}}\,{\rm{by}}\,{\rm{social}}\,{\rm{network}})$$

3 $${x}_{k+1}^{i}(u)={\lambda }^{i}{\bar{x}}_{k}^{i}(u)+(1-{\lambda }^{i}){x}_{0}^{i}\,(u)\,({\rm{Influence}}\,{\rm{of}}\,{\rm{initial}}\,{\rm{beliefs}})$$

where $0\le {x}_{k}^{i}(u)\le 1$ represents the opinion of an agent *i* at time *k* on a certain statement *u*, while ${\hat{x}}_{k}^{i}(u)$ and ${\bar{x}}_{k}^{i}(u)$ are the intermediate aggregation steps. The opinion of an agent on a specific statement being true or false is modeled by a scalar value between zero and one. A value of zero indicates that the given agent strongly believes a specific statement is false, whereas a value of one indicates that the agent believes the statement is true. Similarly, a value of $\frac{1}{2}$ indicates the maximal uncertainty about a statement.

The intermediate aggregated opinion ${\hat{x}}_{k}^{i}(u)$ of agent *i* on statement *u* is formed by using the opinions of the same agent about the other statements *v*. The parameters 0 ≤ *C*_*uv*_ ≤ 1 are compliant with the graph ${\mathscr{T}}$ that models the logic constraints in the sense that *C*_*uv*_ is nonzero if the statement *u* depends on statement *v*, and otherwise *C*_*uv*_ = 0. These parameters represent the strength of the logic constraints, i.e., the influence that an opinion on a statement has on the opinion on other statements. Subsequently, the intermediate aggregated opinion ${\bar{x}}_{k}^{i}(u)$ of agent *i* on statement *u* is formed by combining all the intermediate opinions ${\bar{x}}_{k}^{i}(u)$ of neighboring agents *j*. In this update, the parameters 0 ≤ *A*_*ij*_ ≤ 1 represent the weights that an agent *i* assigns to the information coming from its neighbor *j*, for example *A*_13_ is how agent 1 weights the opinions shared by agent 3. These parameters are compliant with the network ${\mathscr{G}}$ in the sense that if there is an incoming edge to agent *i* from agent *j* in the graph, then the corresponding weight *A*_*ij*_ is nonzero.

Equation (3) indicates that, at time *k* + 1, the new opinion ${x}_{k+1}^{i}(u)$ of agent *i* on statement *u* is obtained as a weighted combination of its intermediate aggregated opinion ${\bar{x}}_{k}^{i}(u)$ at time *k* and its initial opinion ${x}_{0}^{i}(u)$ on statement *u*. The parameter 0 ≤ *λ*^*i*^ ≤ 1 that agent *i* uses models its stubbornness. If *λ*^*i*^ < 1 we say an agent is *stubborn*, where *λ*^*i*^ = 0 indicates that the agent *i* is *maximally closed* to the influence of others. If *λ*^*i*^ = 1, agent *i* is said to be *maximally open* to the influence of others, and *oblivious* if additionally, it is not influenced by stubborn agents. Note that when there are stubborn agents, these can be viewed as leaders in the network, given that their opinions directly influence the final belief values in the network. The concept of leader-follower networks has been previously studied in the consensus and opinion formation literature^18^.

We can group the parameters {*A*_*ij*_} into an *n*-by-*n* matrix *A*, known as the *social influence structure*, and the parameters {*C*_*uv*_} into an *m*-by-*m* matrix *C*, known as the *multi-issues dependent structure*^13^. We assume these matrices are nonnegative. Furthermore, the weights *A*_*ij*_ assigned by an agent *i* to its neighbors *j* sum up to one, i.e., the sum of the entries in each row of the matrix *A* is 1; likewise, the sum of the entries in each row of the matrix *C* is 1. Thus, the matrices *A* and *C* are row-stochastic.

**Figure 1:**
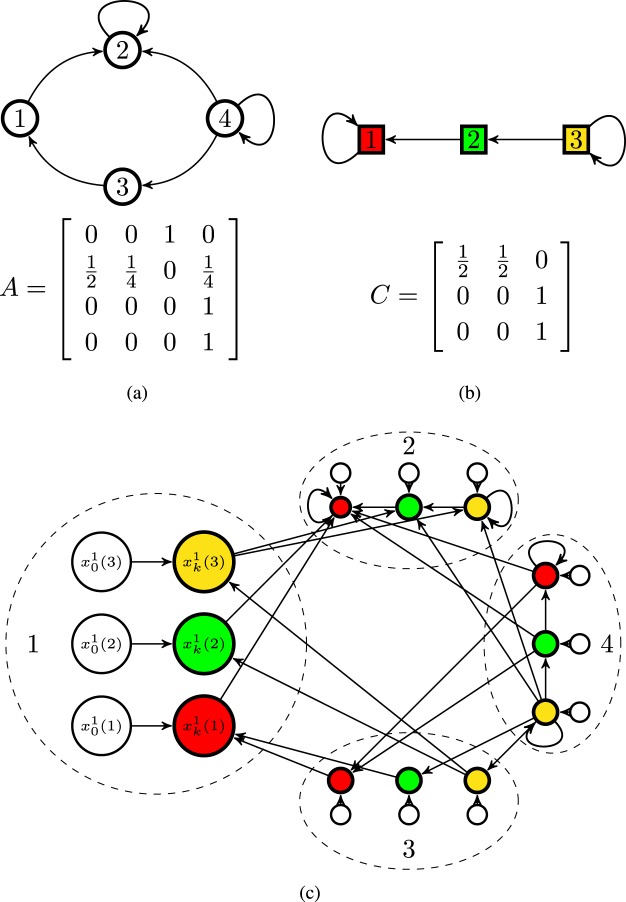
A belief system with 4 agents and 3 truth statements. (**a**) Agents are represented as nodes/circles, numbered from 1 to 4, and the network of influences among them is shown as edges between nodes. The truth statements or topics are color-coded, e.g., the truth statement 1 is represented as a red square. Agent 2 is influenced by its own opinion and agents 4 and 1, agent 1 follows the opinion of agent 3 which in turn follows the opinion of agent 4, agent 4 follows its own opinion only. A possible matrix *A* for this social network is shown below the graph. This indicates that agent 2 assigns a higher weight of $\frac{1}{2}$ to the opinion of agent 1 than the weight it assigns to the opinion of communicated by agent 4. (**b**) The truth statement 1 is influenced by the belief that statement 2 is true, statement 2 directly follows the belief in statement 3. A possible matrix *C* for this set of logic constraints is shown below the graph. The belief that the truth statement 1 is true is influenced (with a weight of $\frac{1}{2}$) by the opinion that the truth statement 2 is true. (**c**) The beliefs system, see Eq. 4, composed by the agent’s interaction graph and the logic constraints.

Figure 1 illustrates a belief system with 4 agents and 3 truth statements, moreover, it gives examples for the choice of the matrices *A* and *C*. Figure 1(c) shows the belief system generated by the network of agents in Fig. 1(a) and the set of logic constraints in Fig. 1(b). This new graph depicted in Fig. 1(c) is much larger than the network of agents or the network of statements taken separately; effectively; it has 2 *nm* nodes. The belief of each agent on each truth statement is a separate node; also, the initial beliefs are separate nodes.

The model of this larger graph of the belief system can be compactly restated as

4 $${x}_{k+1}=P{x}_{k},$$

where *x*_*k*_ ∈ [0, 1]^2*nm*^ is a state that stacks the current beliefs of all agents on all topics alongside with the initial beliefs, i.e.,

$${x}_{k}={[\mathop{\underbrace{{x}_{k}^{1}(1),\ldots ,{x}_{k}^{1}(m)}}\limits_{{\rm{Beliefs}}{\rm{of}}{\rm{Agent}}1},\ldots ,\mathop{\underbrace{{x}_{k}^{n}(1),\ldots ,{x}_{k}^{n}(m)}}\limits_{{\rm{Beliefs}}{\rm{of}}{\rm{Agent}}n},\mathop{\underbrace{{x}_{0}^{1}(1),\ldots ,{x}_{0}^{1}(m)}}\limits_{{\rm{Initial}}{\rm{Beliefs}}{\rm{of}}{\rm{Agent}}1},\ldots ,\mathop{\underbrace{{x}_{0}^{n}(1),\ldots ,{x}_{0}^{n}(m)}}\limits_{{\rm{Initial}}{\rm{Beliefs}}{\rm{of}}{\rm{Agent}}n}]}^{\text{'}}$$

andwhere 0_*nm*_ is a zero matrix of size *n* × *m*, ***I***_*nm*_ is an identity matrix of size *n* × *m*, ⊗ indicates the Kronecker product (see Supplementary Definition 1), Λ is a diagonal matrix with the *i*-th diagonal entry being *λ*^*i*^, and *x*′ denotes the transpose of a vector or matrix *x*. This allows for the definition of the belief system graph ${\mathscr{P}}$, which is compliant with the matrix *P*, where an edge from $\ell$ to *r* exists if ${P}_{r\ell } > 0$.

**Figure 2:**
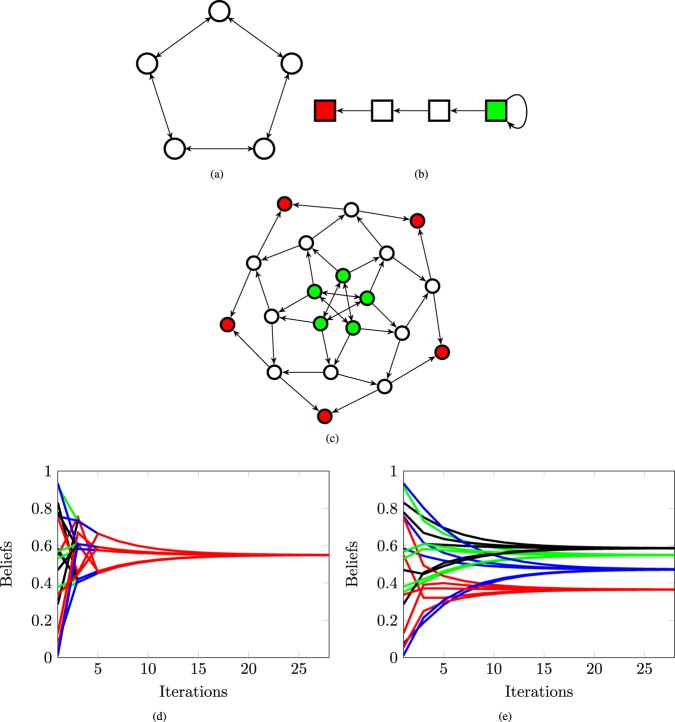
A belief system with agents on a cycle graph and logic constraints on a path graph. (**a**) A network of 5 oblivious agents forming a cycle graph. (**b**) A set of 4 truth statements with logic constraints forming a path graph. (**c**) The belief system graph ${\mathscr{P}}$. (**d**) The belief dynamics with logic constraints. (**e**) The belief dynamics with no logic constraints. The beliefs of all agents have been color coded per truth statement. The agents reach an agreement on each of the truth statements.

Figure 2 shows an example where a network of 5 agents forms a cycle graph, given in Fig. 2(a), a set of 4 logic constraints forms a directed path, given in Fig. 2(b), and *λ*^*i*^ = 1 for all *i*. The belief system graph is shown in Fig. 2(c). Figure 2(d) shows the dynamics of the belief vector as the number of social interactions increases. The opinion on all 4 topics converges to a single value for all agents. Figure 2(e) shows the dynamics of the belief vector when no logic constraints are considered. In this case, the agents reach some agreement on the final value, but this consensual value is different for each of the statements. See Supplementary Fig. 1 for an additional example of the influence of the logic constraints on the resulting belief system and Supplementary Fig. 2 for a variation of the example discussed in Fig. 2 when the network of agents is a complete graph.

Note that once the model in Eq. (2) is determined, one can analyze the the belief system with classical approaches of opinion dynamics involving graph-theoretic properties and algebraic conditions^10,21–24^. Such existing approaches can provide quantitative and qualitative analysis of the belief dynamics in terms of the algebraic and combinatorial properties of the matrix *P*. However, we aim to provide graph-theoretic conditions in terms of the relations generated by the Kronecker product of the social network and network of logic constraints and the individual compositions of open and closed components of each of these graphs.

### When does a belief system converge?

The convergence of the belief system can be stated as a question of the existence of a limit of the beliefs, as the social interactions continue with time. That is, whether there exists a vector of opinions *x*_∞_ such that

$$\mathop{\mathrm{lim}}\limits_{k\to \infty }{x}_{k}=\mathop{\mathrm{lim}}\limits_{k\to \infty }{P}^{k}{x}_{0}={x}_{\infty },$$

for any initial value *x*_0_.

Friedkin *et al*.^12,13^ showed that a belief system with logic constraints will converge to equilibrium if and only if either lim_*k*→∞_(Λ*A*)^*k*^ = 0, or lim_*k*→∞_(Λ*A*)^*k*^ ≠ 0 and lim_*k*→∞_*C*^*k*^ exists. Moreover, if we represent the matrices *A* and Λ with a block structure as

$$\begin{array}{ccc}A=[\begin{array}{ll}{A}^{11} & {A}^{12}\\ 0 & {A}^{22}\end{array}] & {\rm{and}} & {\rm{\Lambda }}=[\begin{array}{ll}{{\rm{\Lambda }}}^{11} & 0\\ 0 & I\end{array}],\end{array}$$

where *A*^22^ is the subgraph of oblivious agents, then the belief system is convergent if and only if lim_*k*→∞_*C*^*k*^ and lim_*k*→∞_(*A*^22^)^*k*^ exists. We next consider how these conditions may be interpreted in terms of the topology of the network of agents and the set of logic constraints.

**Figure 3:**
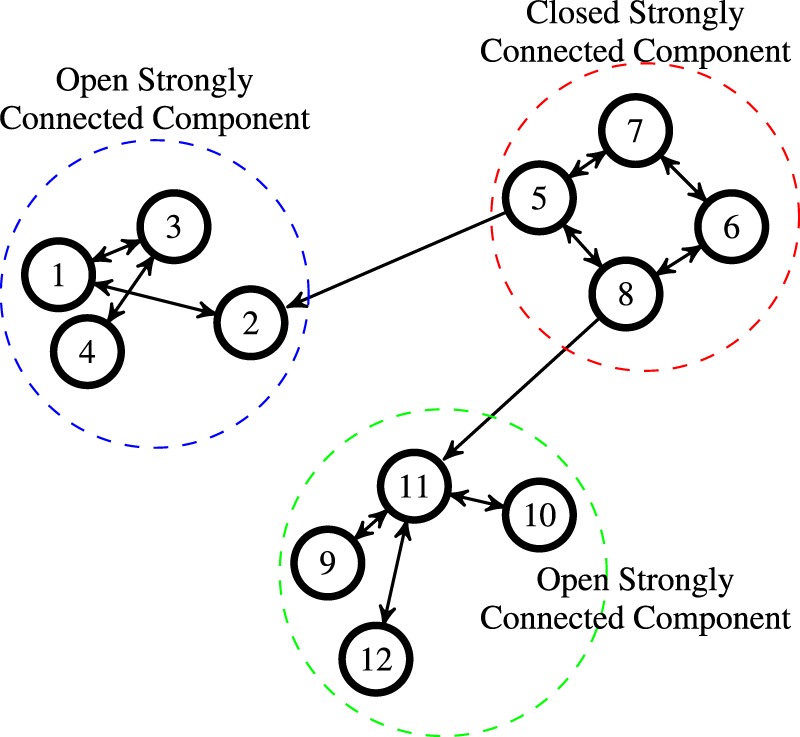
Open and closed strongly connected components of a graph. A graph with 12 nodes and 3 strongly connected components. The strongly connected component composed of nodes 5, 6, 7 and 8 is closed since it has no incoming edges from other components.

The belief system in Eq. (2) converges to equilibrium if and only if every closed strongly connected component of the graph ${\mathscr{P}}$ is aperiodic^4,25^. Recall that a strongly connected component is closed if it has no incoming links from other agents; otherwise, it is called open, see Fig. 3. In general, the set of strongly connected components can be computed efficiently for large-complex networks^26^.

The matrix *P* has two diagonal blocks, one corresponding to the initial beliefs and one involving the product Λ*A* ⊗ *C*. The initial belief nodes are aperiodic closed strongly connected components, each consisting of a single node. Therefore, the diagonal block in *P* corresponding to the initial beliefs induces an aperiodic graph. Moreover, strongly connected components with stubborn agents do not affect the convergence of the belief system. Thus, one can focus on the closed strongly connected components of the graph induced by *A*^22^ ⊗ *C*.

Lemma 1 characterizes the strongly connected components of the product of two graphs. Particularly, it shows that *A*^22^ ⊗ *C* can be written in a block upper triangular form, where each of the blocks in the diagonal is the product of one strongly connected component from the graph induced by *A*^22^ and one from ${\mathscr{T}}$.

#### Lemma 1

*Given two graphs* ${{\mathscr{G}}}_{1}$ *and* ${{\mathscr{G}}}_{2}$, *every strongly connected component of the Kronecker product graph* ${{\mathscr{G}}}_{1}\otimes {{\mathscr{G}}}_{2}$ *is the result of the Kronecker product of a strongly connected component of* ${{\mathscr{G}}}_{1}$ *and a strongly connected component of* ${{\mathscr{G}}}_{2}$.

#### Proof 1

*This proof follows from classical properties of the Kronecker product of graphs*^27^, *and the existence of a topological ordering*^28^. *See the complete proof in the Supplementary Material*: *Supplementary Note* 2.

With Lemma 1 at hand, the following result provides a graph-theoretic condition for the convergence of a belief system, complementing the algebraic criteria derived by Friedkin *et al*.^12,13^.

#### Theorem 2

*The process* in Eq. (2) *converges to equilibrium if an only if every closed strongly connected component of the graph* ${\mathscr{T}}$ *is aperiodic and every closed strongly connected component of the graph* ${\mathscr{G}}$ *composed by oblivious agents only is aperiodic*.

#### Proof 2

*McAndrew*^29^ *showed that the period of a product graph is the lowest common multiple of the periods of the two factor graphs (see* *Supplementary Definition* 2 *and* *Theorem* 1). *If the factor graphs are not coprime, the resulting product graph is a disconnected set of components. Nevertheless, each of the resulting components will have the same period as defined above. Therefore, for a product graph to be aperiodic, we require the factors to be aperiodic as well. Thus, the desired result follows from Lemma 1*.

In Fig. 1, the network of agents has a single closed strongly connected component which consists of the node 4. The network of truth statements also has a single closed strongly connected component, consisting of the node 3. Thus, the belief system will converge to a set of final beliefs. In Fig. 2, the belief system has one closed strongly connected component shown in green with the topology of a cycle graph. This strongly connected component corresponds to the product of the cycle graph and the green node of the logic constraints. The cycle graph is aperiodic if and only if the number of nodes is odd. Thus, if the cycle network of agents has an even number of nodes, the belief system will not converge.

### How long does a belief system take to converge?

We seek to characterize the time required by the process in Eq. (2) to be arbitrarily close to its limiting value in terms of properties of the graphs ${\mathscr{G}}$ and ${\mathscr{T}}$, such as the number of agents and truth statements, and the topology of the graphs.

We provide an estimate on the number of iterations required for the beliefs to be *ε* apart from their final value (assuming they converge). This estimate is expressed in terms of the total variation distance, denoted by ∥⋅∥_*TV*_ (for its definition see the section on Methods). For this we define the convergence time as follows:

$${t}_{{\rm{mix}}}(\varepsilon )=\mathop{{\rm{\min }}}\limits_{k\ge 0}\{k:\mathop{{\rm{\max }}}\limits_{{x}_{0}\in {S}_{1}(2\,nm)}\parallel {x}_{k}-{x}_{\infty }{\parallel }_{TV}\le {\rm{\varepsilon }}\},$$

where *ε* = 1/4 is a common choice, ${S}_{1}(n)=\{x\in {[0,1]}^{n}|\sum _{i=1}^{n}\,{x}_{i}=1\}$ and *x*_*k*_ follows Eq. (2). Informally, *t*_mix_(*ε*) is the minimum number of social interactions required for the belief system to be arbitrarily close to its final value for the worst case initial disagreement.

The dynamics of the belief system in Eq. (2) are closely related to the dynamics of a Markov chain with a transition matrix *P*^30^, specifically, the ergodic properties of a random walk over on the graph ${\mathscr{P}}$. Which are closely related to the spectral properties of the graph Laplacian^20,24^. Particularly, consider a random walk on the state space {1, …, 2 *nm*} which, at time *k* jumps to a random neighbor of its current state. The relation between a random walk on a graph and the convergence properties of systems of the form of the belief system in Eq. (2) has been previously explored in Olshevsky and Tsitsiklis^30^. In both cases, we are interested in the convergence properties of *P*^*k*^ as *k* goes to infinity. If there is a limiting distribution for a Markov chain with transition probability *P*, then the belief system converges. Moreover, bounds on the convergence time based on the mixing properties of this Markov chain provide rates of convergence for the belief system.

Next, we will show that the convergence time of a belief system is proportional to the maximum time required for a random walk, with transition probability matrix *P*, to get *absorbed* into a closed strongly connected component in addition to the time needed for such component to *mix* sufficiently.

#### Lemma 3

*Let* ${\mathscr{P}}$ *be a graph with at least one closed strongly connected component, and assume all its closed strongly connected components are aperiodic. Also, let L be the maximum expected coupling time of a random walk in a closed strongly connected component of* ${\mathscr{P}}$*. Moreover, let H be maximum expected time for a random walk, starting at an arbitrary node, to get absorbed into a closed strongly connected component. Then, for k* ≥ 4(*L* + *H*)log(1/*ε*), *it holds for the belief system described in* *Eq*. (2) *that* ∥*x*_*k*_ − *x*_∞_∥_*TV*_ ≤ *ε*.

#### Proof 3

*See the proof in the Supplementary Material:* *Supplementary Note* 2.

Lemma 3 states that the convergence time of (2) can be bounded by the absorbing time of a random walk on the graph ${\mathscr{P}}$ into a closed strongly connected component, in addition to the mixing time of that particular component. Moreover, the mixing time of a closed strongly connected component can be bounded by its coupling time, i.e., the time needed for two independent random walks, with arbitrary initial points, to intersect^31^.

**Figure 4:**
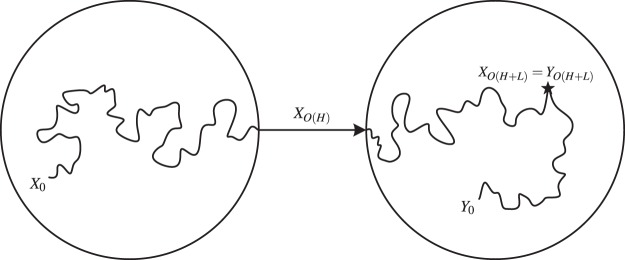
Coupling of two random walks. A random walk starts at *X*_0_ in a transient state and evolves according to some transition matrix *P*; after *O*(*H*) time steps (the absorbing time), it gets absorbed into a closed connected component. Then, after *O*(*L*) time steps (the mixing time) it crosses paths with another random walk *Y*_*k*_ starting at *π* the stationary distribution of *P*. Then after *O*((*L* + *H*)log(1/*ε*)) time steps, the random walk *X*_0_ is arbitrarily close to its limit value. Note that the random walk moves in the opposite direction to the edges in the graph.

**Figure 5:**
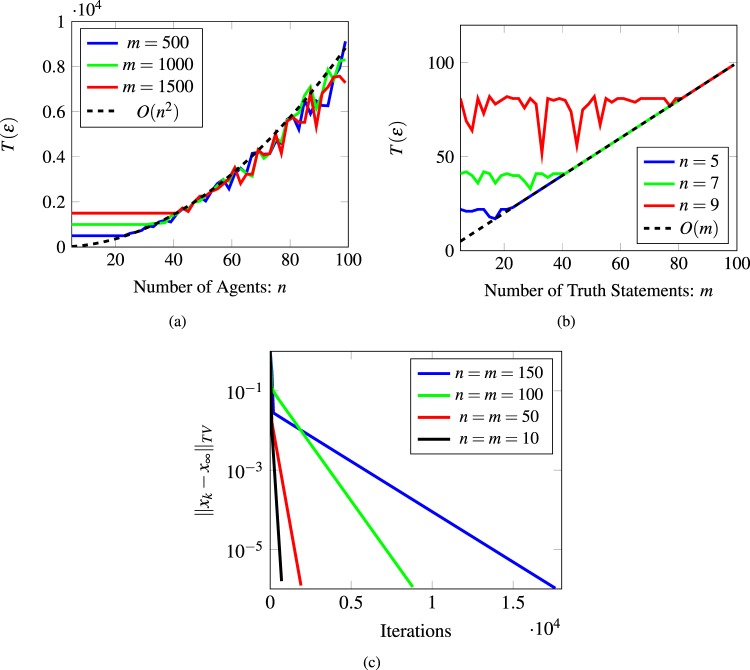
Convergence time for a belief system with an undirected cycle as a social network and a directed path as a network for the logic constraints. (**a**) Varying the number of the agents in the social graph. (**b**) Varying the number of the truth statements for a directed path. (**c**) The exponential convergence rate of the belief system.

Figure 4 illustrates the result in Lemma 3 by considering two random walks *X* and *Y* with the same transition matrix. Assuming the graph ${\mathscr{P}}$ is aperiodic, we denote by *L* the maximum expected mixing time among all closed strongly connected components, and by *H* the maximum expected time to get absorbed into a closed component. Then the belief system will be *ε* close to its limiting distribution after *O*((*L* + *H*)log(1/*ε*)) steps. Therefore, not only do we have an estimate of the convergence time of the belief system in terms of the topology of the graph ${\mathscr{P}}$, but we also know this convergence happens exponentially fast due to the linearity of the involved dynamics. For example, in Fig. 2, the expected absorbing time is of the order of the number of nodes in the path, that is *m*, while the expected mixing time of a cycle graph is of the order of the number of the nodes squared^31–33^, which is *n*^2^ in this example. Thus, the convergence time for the belief system is *O*(max(*n*^2^, *m*)log(1/*ε*)). Figure 5 depicts simulation results for this bound that demonstrate its validity. In particular, Fig. 5(a) shows how the convergence time changes when the number of nodes in the cycle graph increases, while Fig. 5(b) shows how the convergence time changes when the number of truth statements in the directed path graph increases. Moreover, Fig. 5(c) shows that the convergence to the final beliefs is exponentially fast.

Lemma 1 shows that each strongly connected component of the graph ${\mathscr{P}}$ is the product of two such components, one from the graph ${\mathscr{G}}$ and the other from the graph ${\mathscr{T}}$. Consequently, the next lemma shows that the expected mixing (or absorbing) time for a random walk on a product graph is the maximum of the expected mixing (or absorbing) time of the individual factor graphs.

#### Lemma 4

*Consider two aperiodic strongly connected directed graphs* ${{\mathscr{G}}}_{1}$ *and* ${{\mathscr{G}}}_{2}$. *The expected coupling time of two random walks on the graph* ${{\mathscr{G}}}_{1}\otimes {{\mathscr{G}}}_{2}$ *is L* = 8 max{*L*_1_, *L*_2_}, *where L*_*1*_ *and L*_*2*_ *are the expected coupling times for random walks on the graphs* ${{\mathscr{G}}}_{1}$ *and* ${{\mathscr{G}}}_{2}$ *respectively. Similarly, a random walk on an open strongly connected component of a graph* ${{\mathscr{G}}}_{1}\otimes {{\mathscr{G}}}_{2}$ *has an expected absorbing time (into another strongly connected component) of H* = 8 max{*H*_1_, *H*_2_}, *where H*_*1*_ *and H*_*2*_ *are the expected absorbing times for random walks on the graphs* ${{\mathscr{G}}}_{1}$ *and* ${{\mathscr{G}}}_{2}$ *respectively*.

#### Proof 4

*See the proof in the Supplementary Material:* *Supplementary Note* 2.

Lemmas 3 and 4 provide an explicit characterization of the convergence time in terms of the components of the network of agents and the network of logic constraints. Thus allows us to state our graph-theoretic result on the convergence rate of a belief system with logic constraints.

#### Theorem 5

*Assume the process in* Eq. (2) *converges to equilibrium. Moreover, let* ${L}_{{\mathscr{T}}}$ *and* ${H}_{{\mathscr{T}}}$ *be the maximum expected coupling time and maximum absorbing time of the closed aperiodic and strongly connected components of the graph* ${\mathscr{T}}\,$*, and let* ${L}_{{\mathscr{G}}}$ *and* ${H}_{{\mathscr{G}}}$ *be the maximum expected coupling time and absorbing time of a closed aperiodic and strongly connected components of the graph* ${\mathscr{G}}$ *composed by oblivious agents only. Then, for* $k\ge 32({\rm{\max }}\,\{{L}_{{\mathscr{T}}},{L}_{{\mathscr{G}}}\}+\,{\rm{\max }}\,\{{H}_{{\mathscr{T}}},{H}_{{\mathscr{G}}}\})\mathrm{log}(1/\varepsilon )$, *it holds for the belief system in* Eq. (2) *that* ∥*x*_*k*_ − *x*_∞_∥_*TV*_ ≤ *ε*.

#### Proof 5

*The proof follows from Lemmas 1, 3, 4*

Table 1 presents the estimates for the convergence time for belief systems composed of well-known classic graphs, see Supplementary Fig. 3 for plots of some of these common graphs. We use the existing results about the mixing time for these graphs (see Supplementary Table 1 for a detailed list of references on each of the studied graphs) to provide an estimate of the convergence time of the resulting belief system when all agents are oblivious. When available, we present tighter upper bounds for the mixing times on strongly connected components derived with methods other than coupling. Particularly, our method allows the direct characterization of the dynamics of a belief system when large-scale complex networks are involved. For example, we provide convergence time bounds for the case where networks follow random graph models, namely: the geometric random graphs, the Erdös-Rényi random graphs, and the Newman-Watts small-world networks. These graphs are usually considered for their ability to represent the behavior of complex networks encountered in a variety of fields^34–37^ (see Supplementary Fig. 4).

**Table 1:** Convergence time for the belief system with logic constraints for different networks of Agents with *n* nodes and networks of truth statements with *m* nodes.

Network of Agents	Logic Constraints	Convergence Time ≈
Cycle	Directed Path	max(*n*^2^, *m*)
Cycle	Path	max(*n*^2^, *m*^2^)
Dumbbell Graph	Complete Binary Tree	max(*n*^2^, *m*)
*k*-d Hypercube {0,1}^*k*^	Complete Binary Tree	max(*k*log*k*, *m*)
2-d Grid	Star	*n*log*n*
3-d Grid	Two Joined Star	*n*^2/3^log*n*
*k*-d Grid	Star	*k*^2^*n*^2/*k*^log*n*
2-d Torus	2-d Grid	max(*n*^2^, *m*log*m*)
3-d Torus	Star	*n* ^2^
*k*-d Torus	*k*-d Grid	max(*n*^2^*k*log*k*, *k*^2^*m*^2/*k*^log*m*)
Lollipop	Star	*n* ^2^
Dumbbell	Star	*n* ^2^
Eulerian: *d*-degree and expansion	Dumbbell	max(|*E*|^2^, *m*^2^)
Eulerian: *d*-degree, max-degree weights	Dumbbell	max(*n*^2^*d*, *m*^2^)
Lazy Eulerian with degree *d*-degree	Dumbbell	max(*n*|*E*|, *m*^2^)
Lamplighter on *k*-Hypercube	Bolas	max(*k*2^*k*^, *m*^3^)
Lamplighter on (*k*, *n*)-Torus	Bolas	max(*kn*^*k*^, *m*^3^)
Geometric Random on ${\mathbb{R}}$^*d*^: ${{\mathscr{G}}}^{d}(n,r)$	Bolas	max(*r*^−2^log*n*, *m*^3^)
Geometric Random: *r* = Ω(polylog(*n*))	Bolas	max(polylog(*n*), *m*^3^)
Erdös-Rényi: ${\mathscr{G}}(n,c/n)$, *c* > 1	Dumbbell	max(log^2^*n*, *m*^2^)
Erdös-Rényi: ${\mathscr{G}}(n,c/n)$, *c* > 1	Newman-Watts	max(log^2^*n*, log^2^*m*)
Erdös-Rényi: ${\mathscr{G}}(n,(1+\delta )/n)$, *δ*^3^*n* → ∞	Dumbbell	max((1/*δ*^3^)log^2^(*δ*^3^*n*), *m*^2^))
Erdös-Rényi: ${\mathscr{G}}(n,1/n)$	Dumbbell	max(*n*, *m*^2^)
Newman-Watts: ${\mathscr{G}}(n,k,c/n)$, *c* > 0	Path	max(log^2^*n*, *m*^2^)
Expander	Path	*m* ^2^
Any Connected Undirected Graph with Metropolis Weights	Expander	*n* ^2^
Any Connected Undirected Graph	Expander	$|E|{\rm{diam}}({\mathscr{G}})$

The approximated maximum expected convergence time identified as ≈ should be understood in terms of the order *O*(⋅), that is, an estimate up to constant terms. Additionally, all the estimates provided should be multiplied by the accuracy term log(1/*ε*).

**Figure 6:**
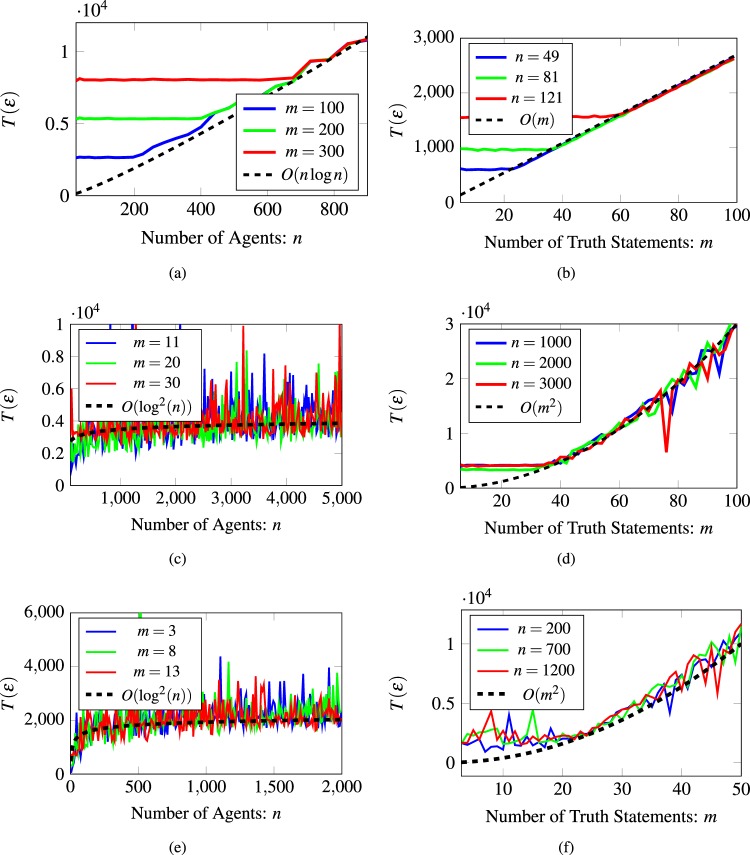
Convergence time or various belief systems. (**a**) Varying the number of agents on a 2d-Grid with fixed the number of truth statements on a star graph. (**b**) Varying the number of truth statements on a star graph with a fixed number of agents on a 2d-Grid. (**c**) Varying the number of agents on a Erdös-Rényi graph with fixed the number of truth statements on a dumbbell graph. (**d**) Varying the number of truth statements on a dumbbell graph with a fixed number of agents on a Erdös-Rényi graph. (**e**) Varying the number of agents on a Newman-Watts small-world graph with fixed the number of truth statements on a path graph. (**f**) Varying the number of truth statements on a path graph with a fixed number of agents on a Newman-Watts small-world graph.

Figure 6 shows experimental results for the convergence time of a belief system for a subset of the graphs given in Table 1. For every pair of graphs, we show how the convergence time increases as the number of agents or the number of truth statements change. One can particularly observe the maximum-like behavior on the convergence time as predicted by the theoretical bounds in Theorem 5. See Supplementary Figs 5 and 7 for additional numerical results on other combinations of graphs from Table 1, and Supplementary Figs 6 and 8 for their linear convergence rates, respectively.

Finally, the next theorem describes how the existence of a clique of a well-connected subset of nodes can guarantee fast mixing of a random walk on a graph.

#### Theorem 6

*Consider a random walk on a connected undirected and static graph* ${\mathscr{G}}=(V,E)$ *with* |*V*| = *n nodes, and assume there is a subset* $\bar{V}\subset V$ *with M nodes such that after K steps, the probability of being in any node in* $\bar{V}$ *is at least* $\frac{1}{5M}$. *Then the mixing time of the corresponding Markov chain is of the order O*(*MK* log (1/*ε*)).

#### Proof 6

*The proof follows immediately since any two random walks will intersect with probability* $\frac{1}{M}$ *every K steps*.

### Where does a belief system converge?

So far we have discussed the conditions for convergence of a belief system and the corresponding convergence time. Convergence implies the existence of a vector *x*_∞_ where the set of beliefs settles as the number of interactions increases. Particularly, Proskurnikov and Tempo^25^ characterize the limiting distribution as a solution of

$${X}_{\infty }={\rm{\Lambda }}A{X}_{\infty }C^{\prime} +(I-{\rm{\Lambda }}){X}_{0},$$

which can be intractable to compute when the matrices *A* and *C* are large. We are interested in a characterization of this limit vector that admits a rapid computation of its value.

Lemma 1 shows that one can always group the nodes in the graph ${\mathscr{P}}$ into open and closed strongly connected components. Moreover, their convergence value depends on whether a node is part of a closed or open strongly connected component of the graph. Thus, our result on the convergence value of a belief system will be stated for nodes in closed or open strongly connected components. However, we start by introducing some notation. Define ${x}_{k}^{S}$ as the vector obtained from *x*_*k*_ by taking only the components of *x*_*k*_ corresponding to the nodes in the set *S*. Moreover, let *P*_*S*_ be the minor of the matrix *P* obtained by taking into account only the nodes in the set *S*. Then, *P*_*S*_ corresponds to the transition matrix of an irreducible and aperiodic Markov chain with a stationary distribution *π*_*S*_, where *π*_*S*_′*P*_*S*_ = *π*_*S*_′. The vector *π*_*S*_ is effectively the left-eigenvector of the matrix *P*_*S*_ corresponding to the eigenvalue 1^31^.

Particularly, for nodes in a closed strongly connected component *S*, it follows that ${x}_{k+1}^{S}={P}_{S}{x}_{k}^{S}$. Whereas for open strongly connected component *S*, we have that ${x}_{k+1}^{S}=Z{x}_{k}^{S}+R{x}_{k}^{{S}_{M}}$, where *Z* is strongly connected and substochastic, meaning some rows add up to less than 1, and *R* represents how the incoming nodes to the set *S*, denoted by *S*^*M*^, influence the nodes in *S*.

#### Theorem 7

*Assume the process* in Eq. (2) *converges to equilibrium. Let S be a strongly connected component of the system graph* ${\mathscr{P}}$, *with factors A*_*S*_ *and C*_*S*_, *i.e*., *P*_*S*_ = *A*_*S*_ ⊗ *C*_*S*_. *If S is closed, then*,

$$\mathop{\mathrm{lim}}\limits_{k\to \infty }{x}_{k}^{i}=({\pi }_{{A}_{S}}\otimes {\pi }_{{C}_{S}})^{\prime} ({x}_{0}^{{A}_{S}}\otimes {x}_{0}^{{C}_{S}})\,\,\forall i\in S.$$

*Moreover, if S is open with edges coming from the set of nodes S*^*M*^, *then*

$$\mathop{\mathrm{lim}}\limits_{k\to \infty }{x}_{k}^{i}=\sum _{j\in {S}^{M}}\,{p}_{ij}{x}_{\infty }^{j}\,\,\forall i\in S,$$

*where p*_*ij*_ *is the probability of absorption of a random walk starting at node i into a node j* ∈ *S*^*M*^ *with limiting value* ${x}_{\infty }^{j}$.

#### Proof 7

*It follows form Lemma 1 that every strongly connected component of* ${\mathscr{P}}$ *is the product of two strongly connected components, one from the network of agents and one from the logic constraint network. Thus*, *P*_*S*_ = *A*_*S*_ ⊗ *C*_*S*_ *for some matrices A*_*S*_ *and C*_*S*_ *(sub-matrices of A and C respectively), which implies that* ${\pi }_{S}={\pi }_{{A}_{S}}\otimes {\pi }_{{C}_{S}}$, *i.e., the vectors* ${\pi }_{S}^{A}$ *and* ${\pi }_{S}^{C}$ *are the corresponding left eigenvalues of the factor components of P*_*S*_ *associated with the eigenvalue 1*.

*On the other hand, without loss of generality, assume that* ${\mathrm{lim}}_{k\to \infty }{x}_{k}^{{S}_{M}}={x}_{\infty }^{{S}_{m}}$. *Therefore, we can analyze the dynamics in the open strongly connected component S as follows: Initially define the following two systems*

$$\begin{array}{ccc}{\bar{x}}_{k+1}^{S}=Z{\bar{x}}_{k}^{S}+R{x}_{\infty }^{{S}_{M}}, & and & {x}_{k+1}^{S}=Z{x}_{k}^{S}+R{x}_{k}^{{S}_{M}}.\end{array}$$

*It follows that*

$$\mathop{\mathrm{lim}}\limits_{k\to \infty }({\bar{x}}_{k+1}^{S}-{x}_{k+1}^{S})=Z\mathop{\mathrm{lim}}\limits_{k\to \infty }({\bar{x}}_{k}^{S}-{x}_{k}^{S})+R\mathop{\mathrm{lim}}\limits_{k\to \infty }({x}_{\infty }^{{S}_{M}}-{x}_{k}^{{S}_{M}})=Z\mathop{\mathrm{lim}}\limits_{k\to \infty }({\bar{x}}_{k}^{S}-{x}_{k}^{S}).$$

*Moreover, given that Z is substochastic, the magnitude of its eigenvalues are strictly less than 1 and I* − *Z is invertible. Thus, we can conclude that* ${\mathrm{lim}}_{k\to \infty }{\bar{x}}_{k}^{S}={\mathrm{lim}}_{k\to \infty }{x}_{k}^{S}$.

*Stacking the vector* ${\bar{x}}_{k}^{S}$ and ${x}_{\infty }^{{S}_{M}}$ *into a single vector we obtain the following recursion:*

$$\begin{array}{ccc}[\begin{array}{c}{\bar{x}}_{k+1}^{S}\\ {x}_{\infty }^{{S}_{M}}\end{array}]={Q}_{S}[\begin{array}{c}{\bar{x}}_{k}^{S}\\ {x}_{\infty }^{{S}_{M}}\end{array}], & where & {Q}_{S}=[\begin{array}{ll}Z & R\\ 0 & I\end{array}].\end{array}$$

*Thus, in order to find the limit value of the set of beliefs in S we can focus on the analysis of the powers of the matrix Q*^*S*^.

We have that

$$\mathop{\mathrm{lim}}\limits_{k\to \infty }{Q}_{S}^{k}=[\begin{array}{ll}0 & NR\\ 0 & I\end{array}],$$

*where N* = *I* + *Z* + *Z*^2^ +…= (1 − *Z*)^−1^. *The matrix NR is the absorbing probability matrix, see Chapter 3 in Kemeny and Snell*^38^, *where* ${p}_{ij}\triangleq {[NR]}_{ij}$ *is the probability of being absorbed by into the node j* ∈ *S*^*M*^ *starting from node i* ∈ *S*. *Moreover, it follows that for any node i* ∈ *S*

$$\mathop{\mathrm{lim}}\limits_{k\to \infty }{x}_{k}^{i}=\sum _{j\in {S}^{M}}\,{p}_{ij}{x}_{\infty }^{j}.$$

Theorem 7 shows that the final beliefs of those nodes in closed strongly connected components are a weighted average of the initial beliefs in that component, and the weights (sometimes referred to as the social power) are determined by the product of the left-eigenvectors of the factors *A*_*S*_ and *C*_*S*_. On the other hand, the limiting value of nodes in an open strongly connected components is a convex combination of the limiting values of the nodes from incoming edges. Moreover, their weights are defined by the absorption probabilities.

### Numerical analysis of social networks

**Figure 7:**
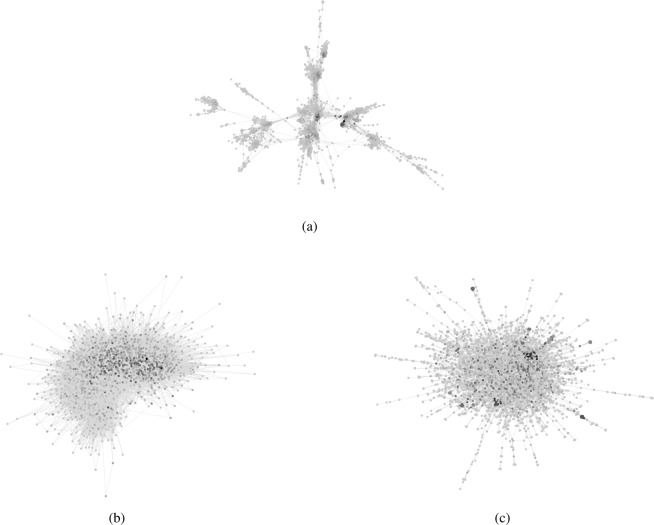
Large-Scale complex networks from the Stanford Network Analysis Project (SNAP). (**a**) The ego-Facebook, nodes are anonymized users from Facebook and edges indicate friendship status between them. (**b**) The wiki-Vote graph, each node represents a Wikipedia administrator and an directed edge represents a vote used for promoting a user to admin status. (**c**) The ca-GrQc graph is a collaboration network from arXiv authors with papers submitted to the General Relativity and Quantum Cosmology category, edges indicated co-authorship of a manuscript. The gray scale in the node colors shows the relative social power according to the left-eigenvector corresponding to the eigenvalue 1.

Next, we provide a numerical analysis for the evolution of belief systems with social network structures from *large-scale networks* in the Stanford Network Analysis Project (SNAP)^39^, see Fig. 7, and logic constraints built from random graph generating models. Random graph generating models, such at the Erdös-Rényi graphs, the Newman-Watts graph, and the geometric random graphs, have been proposed to model the dynamics and the properties of real large-scale complex networks, for example, relatively fast mixing or linear convergence of the beliefs. We use the wiki-Vote^40^, ca-GrQc^41^, and ego-Facebook^42^ graphs as social networks and a binary tree, a Newman-Watts graph, and an Erdös-Rényi graphs as logic constraints.

The wiki-Vote network represents the aggregation of 2794 elections where 7115 Wikipedia contributors assign votes to each other to select administrators. This generates a directed social network where the edges are the votes given by the users. The ca-GrQc network represents the general relativity and quantum cosmology collaboration network for e-prints from arXiv. The nodes are composed of 5242 authors, and edges represent co-authorship of a manuscript between two authors. Finally, the ego-Facebook network represents an anonymized set of Facebook users as nodes and edges indicate friendships among them in the Facebook platform. Table 2 shows the description of the networks used. In the three cases, we select the largest strongly connected component of the graph and use it as a representative of the network structure and the mixing properties of the graph. Furthermore, we assume that the agents use equal weights for all their (in)neighbors.

**Table 2:** Datasets of large-scale networks.

Graph	Nodes	Edges	Type	Upper Bound on Mixing Time	Description
wiki-Vote^40^	1300	103663	Directed	145	Wikipedia who-votes-on-whom network
ca-GrQc^41^	4158	13428	Undirected	12308	Collaboration network of arXiv General Relativity
ego-Facebook^42^	3927	88234	Undirected	53546	Social circles from Facebook

Description, number of nodes, number of edges, simulated mixing time and an upper bound on the mixing time of the three datasets used in the numerical analysis. The upper bound on the mixing time is computed from the second largest eigenvalue bound in Eq. (5).

**Figure 8:**
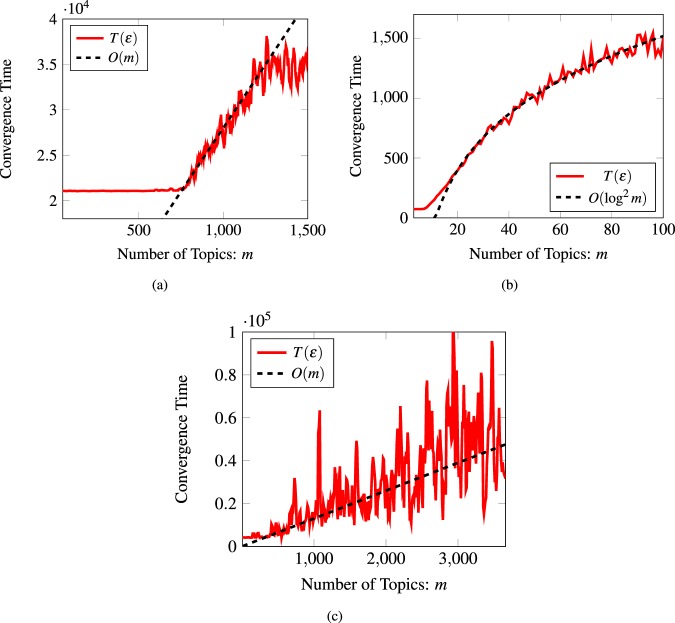
Convergence time of a belief system over a large-scale complex network. (**a**) The social network is the ego-Facebook graph and the logic constraints form a complete binary tree with an increasing number of topics. (**b**) The social network is the wiki-Vote graph and the logic constraints form Newman-Watts small-world graph with an increasing number of topics. (**c**) The social network is the ca-GrQc arXiv collaboration graph, and the logic constraints form an Erdős-Rényi graph with an increasing number of topics.

**Figure 9:**
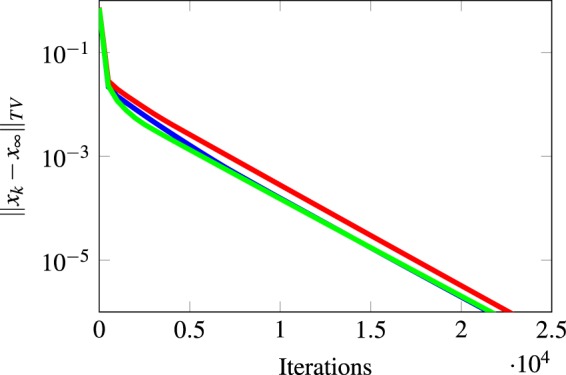
Total variation distance between the beliefs and its limiting value as the number of iteration increases. Results are shown for a particular subset of randomly selected agents.

Figure 8 shows the convergence time of a belief system when the network of agents is each the three large-scale complex networks described in Table 2. Figure 8 considers a simplified scenario where a single closed strongly connected component composes the social network of agents and the network of logic constraints. Therefore, absorbing time is effectively zero and the mixing time of the belief system is the maximum between the mixing time of the social network and the mixing time of the network of logic constraints. Convergence is guaranteed since both networks are taken to be aperiodic by introducing positive self-weights to every agent. Results show that the predicted maximum type behavior holds; that is, the convergence time of the belief system is upper bounded by the maximum mixing time of a random walk on the graph of agents and the graph of logic constraints. The convergence time remains constant and of the order of the convergence time of the network of agents, until the mixing time of the network formed by the logic constraints is larger. Then, the total convergence time increases based on the specific topology of the graph of logic constraints. Figure 9 shows the exponential convergence rate of the belief system described in Fig. 8.

**Figure 10:**
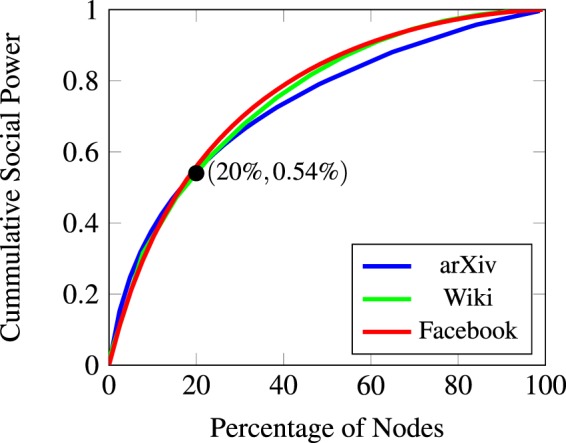
Cumulative social power of the agents. Each of the nodes in the graphs considered has some weight in the final value achieved by the belief system. In all three cases, the 20% most important nodes account for 50% of the final value.

Figure 10 shows the cumulative influence of the nodes in each of the graphs, i.e., the weight an ordered subset of the nodes has on the final value of the beliefs. In this case, since we are considering a single strongly connected component, the weights are determined by the left-eigenvalue of the weight matrix corresponding to the eigenvector 1.

## Discussion

In a recent paper, Friedkin *et al*.^12^ proposed a new model that integrates logic constraints into the evolution opinions of a group of agents in a belief system. Logic constraints among truth statements have a significant impact on the evolution of opinion dynamics. Such restrictions can be modeled as graphs that represent the favorable or unfavorable influence the beliefs on specific topics have on others. Starting from this context, we have here approached this model from its extended representation of a belief system, where opinions of all agents on all topics as well as their corresponding initial values are nodes in a larger graph. This larger graph is composed of the Kronecker product of the graphs corresponding to the network of agents and the network of logical constraints respectively.

In this study, we have provided graph-theoretic arguments for the characterization of the convergence properties of such opinion dynamic models based on extensive existing knowledge of convergence and mixing time of random walks on graphs using the theory of Markov chains. We have shown that convergence occurs if every strongly connected component of the network of logic constraints is aperiodic and every strongly connected component of oblivious agents is aperiodic as well. Moreover, to be arbitrarily close to their limiting value we require *O*((*L* + *H*)log(1/*ε*)) time steps. The parameter *L* is the maximum coupling time for a random walk among the closed strongly connected components of the product graph, and *H* is the maximum time required for a random walk, that starts in an open component, to get absorbed by a closed component. Our analysis applies to broad classes of networks of agents and logic constraints for which we have provided bounds regarding the number of nodes in the graphs. Finally, we show that the limiting opinion value is a convex combination of the nodes in the closed strongly connected components and this convergence happens exponentially fast.

Our framework offers analytical tools that deepen our abilities for modeling, control and synthesis of complex network systems, mainly human-made, and can inspire further research in domains where opinion formation and networks interact naturally, such as neuroscience and social sciences. Finally, extending this analysis to other opinion formation models that use different aggregating strategies may require further study of Markov processes and random walks.

## Methods

### Directed graphs

We define a directed graph ${\mathscr{G}}$ as a set of nodes *V* and a set of edges *E* where the elements of *E* are ordered pairs (*j*, *i*) with *i*, *j* ∈ *V*^29^. A path **P** of ${\mathscr{G}}$ is a finite sequence ${\{{p}_{i}\}}_{i=0}^{l}$ such that (*p*_*i*_, *p*_*i*_ + _1_) ∈ *E* for 0 ≤ *i* ≤ *l* − 1. Moreover, define *n*(**P**) as the number of edges in the path **P**. A pair of nodes (*i*, *j*) are *strongly connected* if there is a path from *i* to *j* and from *j* to *i*. We say a directed graph ${\mathscr{G}}$ is *strongly connected* if each pair of nodes of ${\mathscr{G}}$ are strongly connected. A cycle **C** of a graph ${\mathscr{G}}$ is a path **P** such that *p*_0_ = *p*_*l*_, i.e., the start and end nodes of the path are the same. We denote the *period* of a directed graph as $d({\mathscr{G}})$, and define it as the greatest common divisor of the length of all cycles in the graph ${\mathscr{G}}$. The adjacency matrix *W* of a graph ${\mathscr{G}}$ is defined as the square matrix whose elements represent whether pairs of vertices are adjacent or not in ${\mathscr{G}}$. For example [*W*]_*ij*_ = −1 if (*i*, *j*) ∈ *E*, [*W*]_*ii*_ = deg(*i*) and [*W*]_*ij*_ = 0 otherwise, where deg(*i*) is the degree or number of neighbors of the node *i*.

### Random walks, mixing and Markov chains

Consider a finite directed graph ${\mathscr{G}}=(V,E)$ composed by a set *V* of nodes with a set of edges *E* and a compliant associated row-stochastic matrix *P*, called the transition matrix. A random walk on the graph ${\mathscr{G}}$ is the event of a token moving from one node to an out-neighbor according to some probability distribution determined by the transition matrix. The dynamics of the random walk are modeled a Markov chain $X={({X}_{k})}_{0}^{\infty }$ such that ${\mathbb{P}}${*X*_*k* + 1_ = *j*|*X*_*k*_ = *i*} = *P*(*i*, *j*) with *i*, *j* ∈ *V*. This Markov chain is called *ergodic* if it is irreducible and aperiodic. For an ergodic Markov chain, there exists a unique stationary distribution *π*, which describes the probability that a random walk visits a particular node in the graph as the time goes to infinity, that is ${\mathbb{P}}${*X*_*k*_ = *j*} → *π*_*j*_ as *k* → ∞. The stationary distribution is invariant for the transition matrix, that is *π*′*P* = *π*′. It follows that the convergence to the stationary distribution of a random walk reduces to analyzing powers of *P* (Theorem 4.9 in Levin *et al*.^31^).

The distance to stationarity at a time *k*, i.e., after *k* transitions of the Markov chain, or *k* steps in the random walk, is defined as

$$d(k)=\mathop{{\rm{\max }}}\limits_{x\in {\rm{\Omega }}}\parallel {P}^{k}(x,\cdot \,)-\pi {\parallel }_{TV},$$

where ∥*μ* − *ν*∥_*TV*_ is the *total variation distance* between two probability distributions *μ* and *ν*, defined as

$$\parallel \mu -\nu {\parallel }_{TV}=\mathop{{\rm{\sup }}}\limits_{{\rm{events}}\,A\in  {\mathcal F} }|\mu (A)-\nu (A)|.$$

Moreover, the mixing time of the Markov chain is

$${t}_{{\rm{mix}}}(\varepsilon )=\mathop{{\rm{\min }}}\limits_{k\ge 0}\{k:d(k)\le \varepsilon \},$$

and we say the Markov chain has (relatively) rapid mixing if ${t}_{{\rm{mix}}}(\varepsilon )={\rm{poly}}(\mathrm{log}n,\,\mathrm{log}\,(\frac{1}{\varepsilon }))$, i.e., polynomial relations in the terms log*n* and log(1/*ε*). Finally, the mixing time can be bounded in terms of the left eigenvalues of the matrix *P* as

5 $$\frac{{\lambda }_{2}}{2(1-{\lambda }_{2})}\,\mathrm{log}(\frac{1}{2\varepsilon })\le {t}_{{\rm{mix}}}(\varepsilon )\le \frac{\mathrm{log}\,n+\,\mathrm{log}(1/\varepsilon )}{1-{\lambda }_{2}},$$

where *λ*_2_ is the left-eigenvalue of the transition matrix *P* with the largest abstolute value^43^.

### The coupling method

The technical advances in this paper are mostly made by using the coupling method, which is a way to bound the mixing time of Markov chains. Consider two independent Markov chains $X={({X}_{k})}_{0}^{\infty }$ and $Y={({Y}_{k})}_{0}^{\infty }$, with the same transition matrix *P*. Then, define the *coupling time K* as the smallest *k* such that *X*_*k*_ = *Y*_*k*_, that is, *K* = min_*k*≥0_{*X*_*k*_ = *Y*_*k*_}. Note that *K* is a random variable and it depends on *P* as well as the initial distributions of the processes *X* and *Y*. Finally, define the quantity *L* as the maximum expected coupling time of a Markov chain with transition matrix *P* over all possible initial distributions of the processes *X* and *Y*, i.e.,

$$\begin{array}{ccc}L=\mathop{{\rm{\max }}}\limits_{u,v}{\mathbb{E}}[K] & {\rm{where}} & {X}_{0}=u\,\,\mathrm{and}\,\,{Y}_{0}=v.\end{array}$$

In words, *L* is the maximum expected time it takes for two random walks, with the same transition matrix and arbitrary initial states, to intersect. If *X* starts from a distribution *π*, and *Y* from some other arbitrary stochastic vector *v*, we *couple* the processes *Y* and *X* by defining a new process *W* such that

$${W}_{k}=(\begin{array}{ll}{Y}_{k}, & \mathrm{if}\,\,k < K\\ {X}_{k}, & \mathrm{if}\,\,k\ge K\end{array}$$

The key insight of the coupling method is that *W*_*k*_ is identically distributed to *X*_*k*_; this follows by conditioning on the events *K* ≤ *k* and *K* > *k*. Therefore, questions about the distribution of *X*_*k*_ can be solved by considering *W*_*k*_ instead.

By starting the chain *X*_*k*_ in the stationary distribution, these considerations imply that

$$\parallel v^{\prime} {P}^{k}-\pi {\parallel }_{TV}\le {\mathbb{P}}\{K > k\},$$

because if *K* ≤ *k* then *W*_*k*_ = *Y*_*k*_; for more details, see Lindvall^44^. Thus, it follows by the Markov inequality that

$$\parallel v^{\prime} {P}^{k}-\pi {\parallel }_{TV}\le \frac{{\mathbb{E}}[K]}{k}.$$

Setting *k* = 2${\mathbb{E}}$[*K*] implies that ∥*v*′*P*^*k*^ − *π*∥_*TV*_ ≤ 1/2. Thus, it follows that after *T* = *O*(*L*log(1/*ε*)) steps, it holds that ∥*v*^*T*^*P*^*T*^ − *π*∥_1_ ≤ *ε*, for any *v*, and *π* being the stationary distribution of the Markov chain. Since ||*p* − *q*||_*TV*_ = (1/2)||*p* − *q*||_1_^31^, the same applies to the quantity ∥*v*′*P*^*k*^ − *π*∥_1_.

The coupling method is the primary technical tool we use in this work. In Supplementary Note 3, we use the coupling method to bound the convergence time of Eq. (1) in terms of the coupling times on the underlying social network and on the logic constraint graph. Because coupling time over the Kronecker product is, up to a multiplicative constant, the maximum of the coupling times, this allows us to analyze the effect of the social network and logic constraint graph on convergence time separately.

## Supplementary information

Supplementary Material: Graph-Theoretic Analysis of Belief System Dynamics under Logic Constraints
