# Supplementary material for: Graph-Theoretic Analysis of Belief System Dynamics under Logic Constraints
Source: Sci Rep. 2019 Jun 20;9:8843. doi: 10.1038/s41598-019-45076-4 (PMC6586829; doi:10.1038/s41598-019-45076-4)
Supplement: Supplementary file 1 — Supplementary Material: Graph-Theoretic Analysis of Belief System Dynamics under Logic Constraints [file 41598_2019_45076_MOESM1_ESM.pdf]

Supplementary Material: Graph-Theoretic Analysis of Belief System Dynamics under Logic Constraints

Angelia Nedić[1], Alex Olshevsky[2], and César A. Uribe[3,*]

[1]Arizona State University, ECEE Department, Tempe, 85287, USA
[2]Boston University, ECE Department and Division of Systems Engineering, Boston, 02215, USA
[3]Massachusetts Institute of Technology, Laboratory for Information and Decision Systems (LIDS), and the Institute for Data, Systems, and Society (IDSS), Cambridge, 02139, USA
[*]cauribe@mit.edu

ABSTRACT

This is the supplementary material for the paper: " Graph-Theoretic Analysis of Belief System Dynamics under Logic Constraints."

Supplementary Note 1: The Kronecker Product of Graphs

In this note, we define the Kronecker product of two matrices and the Kronecker product of two graphs. Also, we show some of the properties we will use in the proof of our main results regarding convergence, convergence time and limiting value of belief systems.

Definition 1 ([1,2]) *Let A be a $m \times n$ matrix, and C be a $p \times q$ matrix, the* ***Kronecker product*** *$A \otimes C$ is the $mp \times nq$ matrix defined as:*

$$A \otimes C = \begin{bmatrix} a_{11}C & \dots & a_{1n}C \\ \vdots & \ddots & \vdots \\ a_{m1}C & \dots & a_{mn}C \end{bmatrix},$$

or explicitly

$$A \otimes C = \begin{bmatrix} a_{11}\begin{bmatrix} c_{11} & \dots & c_{1q} \\ \vdots & \ddots & \vdots \\ c_{p1} & \dots & c_{pq} \end{bmatrix} & \dots & a_{1n}\begin{bmatrix} c_{11} & \dots & c_{1q} \\ \vdots & \ddots & \vdots \\ c_{p1} & \dots & c_{pq} \end{bmatrix} \\ \vdots & \ddots & \vdots \\ a_{m1}\begin{bmatrix} c_{11} & \dots & c_{1q} \\ \vdots & \ddots & \vdots \\ c_{p1} & \dots & c_{pq} \end{bmatrix} & \dots & a_{mn}\begin{bmatrix} c_{11} & \dots & c_{1q} \\ \vdots & \ddots & \vdots \\ c_{p1} & \dots & c_{pq} \end{bmatrix} \end{bmatrix}$$

$$
= \begin{bmatrix}
a_{11}c_{11} & \dots & a_{11}c_{1q} & \dots & a_{1n}c_{11} & \dots & a_{1n}c_{1q} \\
\vdots & \ddots & \vdots & & \vdots & \ddots & \vdots \\
a_{11}c_{p1} & \dots & a_{11}c_{pq} & \dots & a_{1n}c_{p1} & \dots & a_{1n}c_{pq} \\
\vdots & & \vdots & & \vdots & & \vdots \\
\vdots & & \vdots & & \vdots & & \vdots \\
a_{m1}c_{11} & \dots & a_{m1}c_{1q} & \dots & a_{mn}c_{11} & \dots & a_{mn}c_{1q} \\
\vdots & \ddots & \vdots & & \vdots & \ddots & \vdots \\
a_{m1}c_{p1} & \dots & a_{m1}c_{pq} & \dots & a_{mn}c_{p1} & \dots & a_{mn}c_{pq}
\end{bmatrix}.
$$

Next, we will enumerate some useful properties of the Kronecker product.

1. Bilinearity and associativity: for matrices A, B and C, and a scalar k, it holds:

$$
\begin{aligned}
A \otimes (B+C) &= A \otimes B + A \otimes C \\
(A+B) \otimes C &= A \otimes C + B \otimes C \\
(kA) \otimes C &= A \otimes (kB) = k(A \otimes B) \\
(A \otimes B) \otimes C &= A \otimes (B \otimes C).
\end{aligned}
$$

2. Non-Commutative: In general $A \otimes B \neq B \otimes A$. However, there exist commutation matrices P and Q such that:

$$A \otimes B = P(B \otimes A)Q,$$

   and if A and B are square matrices then $P = Q'$.

3. Mixed-product property: for matrices A, B, C and D:

$$(A \otimes B)(C \otimes D) = (AC) \otimes (BD).$$

Next, we introduce the Kronecker product of graphs and some of its properties.

Definition 2 ([1] Definition 1) *The Kronecker (also known as categorical, direct, cardinal, relational, tensor, weak direct or conjunction) product* $\mathscr{G} = \mathscr{G}_1 \otimes \mathscr{G}_2$ *of two graphs* $\mathscr{G}_1 = (V_1, E_1)$ *and* $\mathscr{G}_2 = (V_1, E_1)$ *is a graph* $\mathscr{G} = (V, E)$ *where* $V = V_1 \times V_2$*; and* $(u, u') \to (v, v') \in E$ *if and only if* $u \to v \in E_1$ *and* $u' \to v' \in E_2$*. Moreover, the adjacency matrix of the graph* $\mathscr{G}$ *is the Kronecker product of the adjacency matrices of* $\mathscr{G}_1$ *and* $\mathscr{G}_2$*.*

Theorem 1 ([3] Theorem 1) *Let* $\mathscr{G}$ *and* $\mathscr{H}$ *be strongly connected graphs. Let* $d_1 = d(\mathscr{G})$*,* $d_2 = d(\mathscr{H})$*,* $d_3 = gcd(d_1, d_2)$ *and* $D = lcm(d1, d2)$*. Then, the number of components in* $\mathscr{G} \otimes \mathscr{H}$ *is* d_3*. Moreover, for any component* $\mathscr{B}$ *of* $\mathscr{G} \otimes \mathscr{H}$*,* $d(\mathscr{B}) = D$*.*

Supplementary Note 2: Main Technical Results

We now describe the proof of our main result, namely the number of interactions required for a belief system to be arbitrarily close to its limiting set of beliefs. We start with a technical lemma about the strongly connected components of the product of two graphs.

Lemma 1 *Given two graphs* $\mathscr{G}_1$ *and* $\mathscr{G}_2$*, every strongly connected component of the Kronecker product graph* $\mathscr{G}_1 \otimes \mathscr{G}_2$ *is the result of the Kronecker product of a strongly connected component of* $\mathscr{G}_1$ *and a strongly connected component of* $\mathscr{G}_2$*.*

Proof 1 *Let A_1 and A_2 denote the adjacency matrices for the graphs $\mathscr{G}_1$ and $\mathscr{G}_2$, respectively. We can construct a condensation of the graph $\mathscr{G}$ by contracting every strongly connected component to a single vertex, resulting in a directed acyclic graph. Thus, a topological ordering is possible (see Cormen et al.*[4] *Section 22.4) and there always exists two permutation matrices P_1 and P_2 such that we can rearrange the matrices A_1 and A_2 into a block upper triangular form where each of the blocks is a strongly connected component, that is*

$$P_1'A_1P_1 = \begin{bmatrix} A_1^1 & * & * & * \\ 0 & A_1^2 & * & * \\ 0 & 0 & \ddots & * \\ 0 & 0 & \dots & A_1^{n_1} \end{bmatrix} \quad \text{and} \quad P_2'A_2P_2 = \begin{bmatrix} A_2^1 & * & * & * \\ 0 & A_2^2 & * & * \\ 0 & 0 & \ddots & * \\ 0 & 0 & \dots & A_2^{n_2} \end{bmatrix}.$$

Moreover, define $P = P_1 \otimes P_2$ and by the properties of the Kronecker product, cf., Definition 1, it follows that

$$(P_1'A_1P_1) \otimes (P_2'A_2P_2) = P'(A_1 \otimes A_2)P,$$

where P is also a permutation matrix and

$$P'(A_1 \otimes A_2)P = \begin{bmatrix} A_1^1 \otimes A_2 & * & * \\ 0 & \ddots & * \\ 0 & \cdots & A_1^{n_1} \otimes A_2 \end{bmatrix}.$$

Finally, by Property 2 in Definition 1 there exists a permutation matrix Q such that

$$Q'(P'(A_1 \otimes A_2)P)Q = \begin{bmatrix} A_2 \otimes A_1^1 & * & * \\ 0 & \ddots & * \\ 0 & \cdots & A_2 \otimes A_1^{n_1} \end{bmatrix}$$
$$= \begin{bmatrix} A_2^1 \otimes A_1^1 & * & * & * & * & * & * \\ 0 & \ddots & * & * & * & * & * \\ 0 & \cdots & A_2^{n_2} \otimes A_1^1 & * & * & * & * \\ 0 & \cdots & 0 & \ddots & * & * & \\ 0 & \cdots & \cdots & 0 & A_2^1 \otimes A_1^{n_1} & * & * \\ 0 & \cdots & \cdots & \cdots & 0 & \ddots & * \\ 0 & \cdots & \cdots & \cdots & \cdots & 0 & A_2^{n_2} \otimes A_1^{n_1} \end{bmatrix}.$$

Therefore, every block in the upper triangular block diagonal form of the product of two adjacency matrices is the product of two strongly connected components, one from each graph.

We are now ready to state our main technical result regarding the expected mixing time of a Markov Chain whose transition probability matrix is a Kronecker product of two stochastic matrices.

Lemma 3 *Let $\mathscr{P}$ be a graph with at least one closed strongly connected component, and assume all its closed strongly connected components are aperiodic. Also, let L be the maximum expected coupling time of a random walk in a closed strongly connected component of $\mathscr{P}$. Moreover, let H be maximum expected time for a random walk, starting at an arbitrary node, to get absorbed into a closed strongly connected component. Then, for $k \geq 4(L+H)\log(1/\varepsilon)$, it holds for the belief system described in equation (**??**) that $\|x_k - x_\infty\|_{TV} \leq \varepsilon$.*

Proof 2 *We use the coupling method to bound the convergence time of the belief system*[5]*. Initially, we show that all opinions x_k^i, such that i lies in a closed strongly connected component, will converge to some stationary point. Thus, in what follows we will find the required time to reach some ε-consensus via coupling arguments, which in turn will provide the required time for a belief system to be ε close to its stationary distribution.*

Let i be a node belonging to a closed strongly connected component S and let P_S be the matrix obtained by looking at the minor of P corresponding to entries in S. If S is closed then P_S is row-stochastic, and Perron-Frobenius theory tells us there exists some vector π_S such that

$$\pi_S' P_S = \pi_S'.$$

Now, define two independent random walks $X = (X_k)_0^\infty$ and $Y = (Y_n)_0^\infty$ with the same transition matrix P_S. X starts from the distribution π_S, and Y from some other arbitrary stochastic vector v. Moreover, couple the processes Y and X by defining a new process W such that

$$W_k = \begin{cases} Y_k, & \text{if } k < K, \\ X_k, & \text{if } k \geq K, \end{cases}$$

where $K = \min\{k \geq 0 : Y_k = X_k\}$ is called the coupling time. Each random walk moves according to P_S, so if we correlate them by moving them together after they intersect, we have not changed the fact that, individually, they move according to P_S. With this construction of the coupling[6] *Theorem 5.2, we have that*

$$\|v' P_S^k - \pi_S\|_{TV} \leq \max_v \mathbb{P}\{K > k\},$$

and by the Markov inequality

$$\|v' P_S^k - \pi_S\|_{TV} \leq \frac{\max_v \mathbb{E}[K]}{k}.$$

Therefore, to be at a distance of at most $1/4$ we require $k = 4\max_v \mathbb{E}[K]$. We say the mixing time of the random walk is $4L$ where we have that $L = \max_v \mathbb{E}[K]$ is the maximum expected time it takes for the random walks X and Y in S to intersect. Then, it follows that in order to be ε close to the stationary distribution we require at least $k \geq 4L\log(1/\varepsilon)$ steps[6] *Eq. 4.36, for any v. Therefore, we have shown that x_k^i for i in a closed strongly connected component S converges to $\pi_S' x_0^S$ at a geometric rate. Here x_0^S stacks those x_0^i that belong to S.*

Now, consider the case where i belongs to an open strongly connected component. Let M be the set of states in such connected component. Stacking up x_k^i over i in M into the vector x_k^M, observe that

$$x_{k+1}^M = Z x_k^M + R y_k, \tag{1}$$

where Z is strongly connected and substochastic, meaning some rows add up to less than 1. The entries of y_k come from nodes in other strongly connected components and the matrix R represents how they influence the nodes in M.

Initially, assume that y_k converges and call its limit y_∞. Now, consider a random walk that moves around M according to Z; the moment it steps out of M into another strongly connected component we say it is absorbed by it since it can not return to M.

Let q_k^i be the probability the walk is at state i in M at time k. Then

$$q_{k+1}' = q_k' Z,$$

and let H_i be the expected time to get absorbed into any other strongly connected component, the set of nodes in M is connected to, starting from node i and let

$$H^1 = \max_{i \in M} H_i.$$

If the absorbing strongly connected component is closed, then $H = H^1$. On the other hand, the absorbing strongly connected component will have some other absorbing time H^2, i.e., the time to get absorbed into another strongly connected component. Thus, the total absorbing time H is the sum of the absorbing times of the strongly connected components on the longest path on the condensation of the graph $\mathcal{G}$ from an open strongly connected component to a closed strongly connected component. The condensation of the graph $\mathcal{G}$ is a directed acyclic graph and such path always exist.

By the Markov inequality, regardless of where the random walk starts, the probability that it takes more than $4H$ *iterations to get absorbed is at most* $1/4$*. Thus, for all* $k \geq 4H\log(1/\varepsilon)$ *steps we have that* $\|q_k\|_1 < \varepsilon$*.*

Now, let z_∞ *be the vector that satisfies*

$$z_\infty = Zz_\infty + Ry_\infty, \tag{2}$$

which we know exists since every eigenvalue of Z *must be strictly less than* 1 *(since* $Z^k \to 0$*). If we define*

$$\Delta_k = x_k^M - z_\infty,$$

then subtracting the updates of x_M *and* z_∞*,*

$$\Delta_{k+1} = Z\Delta_k + R(y_k - y_\infty). \tag{3}$$

It follows that Δ_k *goes to zero since we have assumed that* $y_k \to y_\infty$*, and* $Z^k \to 0$*.*

In conclusion, this argument shows that for all $k \geq 4(L+H)\log(1/\varepsilon)$ *steps every node is within* ε *of its limiting value.*

The next lemma states the relation of the coupling and absorbing time for random walks on product graphs. Specifically, it shows a maximum-type behavior where the coupling and absorbing time of the product system is the maximum of coupling and aborning of the factors.

Lemma 4 *Consider two aperiodic strongly connected directed graphs* $\mathscr{G}_1$ *and* $\mathscr{G}_2$*. The expected coupling time of two random walks on the graph* $\mathscr{G}_1 \otimes \mathscr{G}_2$ *is* $L = 8\max\{L_1, L_2\}$*, where* L_1 *and* L_2 *are the expected coupling times for random walks on the graphs* $\mathscr{G}_1$ *and* $\mathscr{G}_2$ *respectively. Similarly, a random walk on an open strongly connected component of a graph* $\mathscr{G}_1 \otimes \mathscr{G}_2$ *has an expected absorbing time (into another strongly connected component) of* $H = 8\max\{H_1, H_2\}$*, where* H_1 *and* H_2 *are the expected absorbing times for random walks on the graphs* $\mathscr{G}_1$ *and* $\mathscr{G}_2$ *respectively.*

Proof 3 *Say both graphs* $\mathscr{G}_1$ *and* $\mathscr{G}_2$ *are aperiodic and strongly connected, their product is also aperiodic and strongly connected and there exists a limiting distribution* π *for a random walk moving on the Kronecker product graph* $\mathscr{G}_1 \otimes \mathscr{G}_2$*.*

Consider a random walk $X = (X_k)_0^\infty$*, on the graph* $\mathscr{G}_1 \otimes \mathscr{G}_2$*, with transition matrix* $A_1 \otimes A_2$ *starting with some arbitrary distribution* v*, where* A_1 *is the transition probability on a random walk on the graph* $\mathscr{G}_1$ *and* A_2 *is the transition probability on a random walk on the graph* $\mathscr{G}_2$*. Moreover, from the definition of the Kronecker product of graphs, we have that the state space of* $\mathscr{G}_1 \otimes \mathscr{G}_2$ *is the Cartesian product* $V = V_1 \times V_2$*, composed by the ordered pairs* (i,j) *for* $i \in V_1$ *and* $j \in V_2$*. Thus, the probability that the random walk* X *jumps from the node* (i,j) *to the node* $(\bar{i},\bar{j})$ *is* $[A_1]_{i,\bar{i}}[A_2]_{j,\bar{j}}$*.*

Following the coupling method, define another random walk $Y = (Y_k)_0^\infty$ *with the same transition matrix* $A_1 \otimes A_2$ *but starting at the stationary distribution* π*. Now, construct an new random walk as follows:*

$$W_k = \begin{cases} Y_k, & \text{if } k < K, \\ X_k, & \text{if } k \geq K, \end{cases}$$

where $K = \min\{k \geq 0 : Y_k = X_k\}$*. Clearly, if the state of the random walk* X *at time* k *is* $X_k = (i_k, j_k)$ *and the state of the random walk* Y *at time* k *is* $Y_k = (\bar{i}_k, \bar{j}_k)$*, then the condition* $Y_k = X_k$ *implies that* $i_k = \bar{i}_k$ *and* $j_k = \bar{j}_k$*. Thus, the coupling time* K *can alternatively be expressed in terms of the two separate conditions* $i_k = \bar{i}_k$ *and* $j_k = \bar{j}_k$*, which in turn represents the coupling conditions for two separate random walks on each individual coordinate where each coordinate represents one of the factor graphs. Therefore, we write the coupling time between the random walks* X *and* Y *as* $K = \min\{k \geq 0 : Y_k = X_k\} = \min\{k \geq 0 : i_k = \bar{i}_k, j_k = \bar{j}_k\}$ *which is equivalent to*

$$\begin{aligned} K &= \min\{k \geq 0 : Y_k = X_k\} \\ &= \min\{k \geq 0 : i_k = \bar{i}_k, j_k = \bar{j}_k\} \\ &= \max\{\min\{k \geq 0 : i_k = \bar{i}_k\}, \min\{k \geq 0 : j_k = \bar{j}_k\}\} \\ &= \max\{K_1, K_2\}, \end{aligned}$$

where K_1 and K_2 are the coupling times for the graphs $\mathscr{G}_1$ and $\mathscr{G}_2$ respectively. Thus,

$$\begin{aligned}\mathbb{P}\{K > k\} &= \mathbb{P}\{\max\{K_1, K_2\} > k\} \\ &\leq \mathbb{P}\{K_1 \geq k\} + \mathbb{P}\{K_2 \geq k\},\end{aligned}$$

where the last inequality follows from the union bound.

Note that given that the initial state of the random walk X is v, the random walks on each of its coordinates have some well defined initial state, $v_1(i) = \sum_{j \in V_2} v((i,j))$ and $v_2(j) = \sum_{i \in V_2} v((i,j))$, where $v_1(i)$ is the probability of staring in node $i \in V_1$, $v_2(j)$ is the probability of starting in node $j \in V_2$, and $v((i,j))$ is the probability of the random walk X to start in the node (i,j).

It follows from Theorem 5.2 in Levin et. al.[6] *that*

$$\begin{aligned}\|v'(A_1 \otimes A_2)_S - \pi\|_{TV} &\leq \max_v \mathbb{P}\{K > k\} \\ &\leq \max_{v_1} \mathbb{P}\{K_1 > k\} + \max_{v_2} \mathbb{P}\{K_2 > k\} \\ &\leq \max_{v_1} \frac{\mathbb{E}[K_1]}{k} + \max_{v_2} \frac{\mathbb{E}[K_2]}{k} \\ &= \max_{v_1} \frac{L_1}{k} + \max_{v_2} \frac{L_2}{k}.\end{aligned}$$

Thus, in order to be at a distance at most $1/4$ from the stationary distribution we require $k \geq 8\max\{L_1, L_2\}$. Moreover, in order to be ε close to the stationary distribution we require at least $k \geq 8\max\{L_1, L_2\}\log(1/\varepsilon)$ steps in the random walk for any initial state v. Finally, the coupling time of X is $L = O(\max\{L_1, L_2\})$.

A similar argument follows for the absorbing time of a random walk on a transient component defined by a product graph requires both coordinates be absorbed individually, thus $H = O(\max\{H_1, H_2\})$.

References

1. Weichsel, P. M. The Kronecker product of graphs. *Proc. Am. Math. Soc.* **13**, 47–52 (1962).

2. Horn, R. A. & Johnson, C. R. *Topics in Matrix Analysis* (Cambridge University Press, 1991).

3. McAndrew, M. H. On the product of directed graphs. *Proc. Am. Math. Soc.* **14**, 600–606 (1963).

4. Cormen, T. H., Leiserson, C. E., Rivest, R. L. & Stein, C. *Introduction to Algorithms, Third Edition* (The MIT Press, 2009), 3rd edn.

5. Lindvall, T. *Lectures on the Coupling Method* (John Wiley & Sons, New York, 1992).

6. Levin, D. A., Peres, Y. & Wilmer, E. L. *Markov Chains and Mixing Times* (American Mathematical Society, Providence, 2009).

7. Penrose, M. *Random Geometric Graphs* (Oxford University Press, 2003).

8. Erdos, P. & Rényi, A. On the evolution of random graphs. *Publ. Math. Inst. Hung. Acad. Sci* **5**, 17–60 (1960).

9. Newman, M. E. & Watts, D. J. Renormalization group analysis of the small-world network model. *Phys. Lett. A* **263**, 341–346 (1999).

10. Ikeda, S., Kubo, I. & Yamashita, M. The hitting and cover times of random walks on finite graphs using local degree information. *Theor. Comput. Sci.* **410**, 94–100 (2009).

11. Beveridge, A. & Wang, M. Exact mixing times for random walks on trees. *Graphs Comb.* **29**, 757–772 (2013).

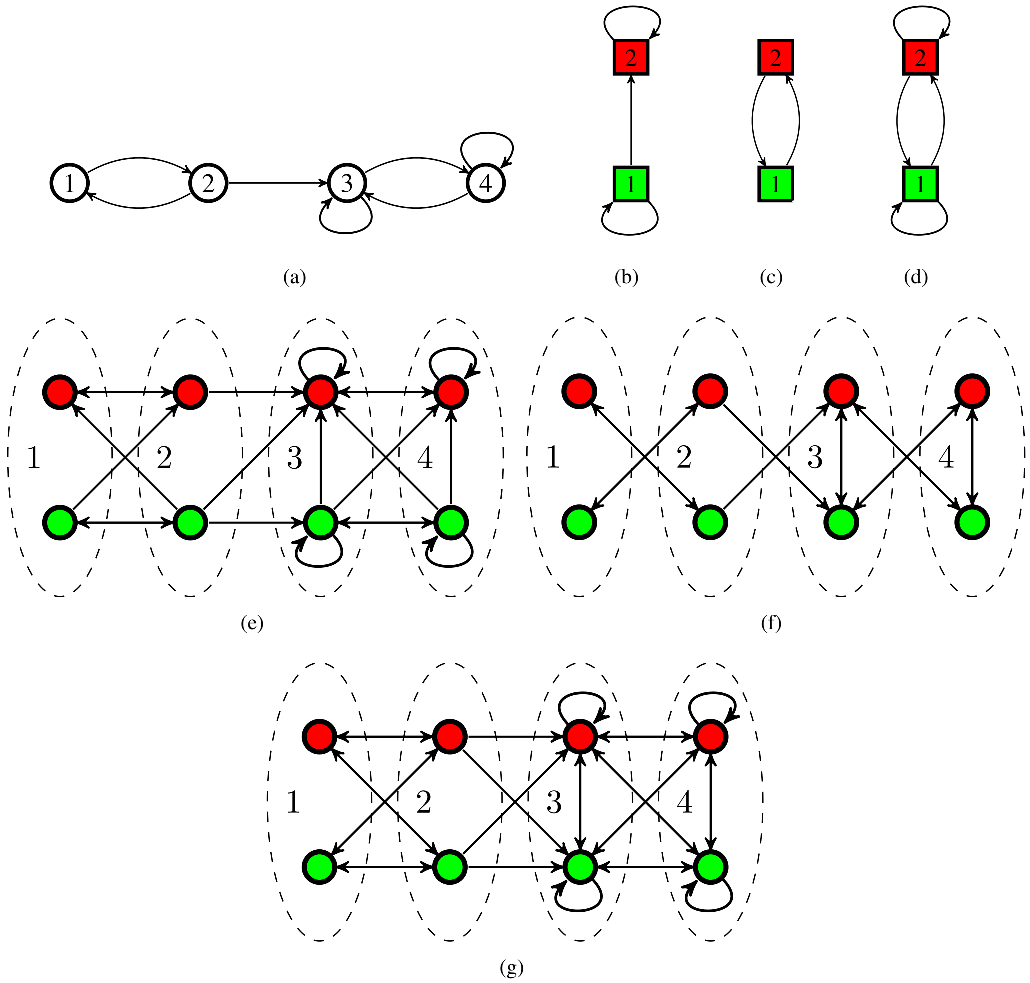

Figure 1. The influence of the logic constraints in the resulting aggregated belief system. (a) The network of agents, where agent 1 follows the opinion of agent 2, agent 2 is influenced by agent 1 and 3, agent 3 is influenced by its own opinion, and the opinion of agent 4 and agent 4 is influenced by agent 3 as well as its own. (b) The opinion on statement 1 is influenced by the belief on statement 2. (c) The opinion on statements 2 and 1 follow each other. (d) The opinion on statements 2 and 1 influence each other (e-g) The belief systems with the network of agents in (a) and logic constraints in (b-d).

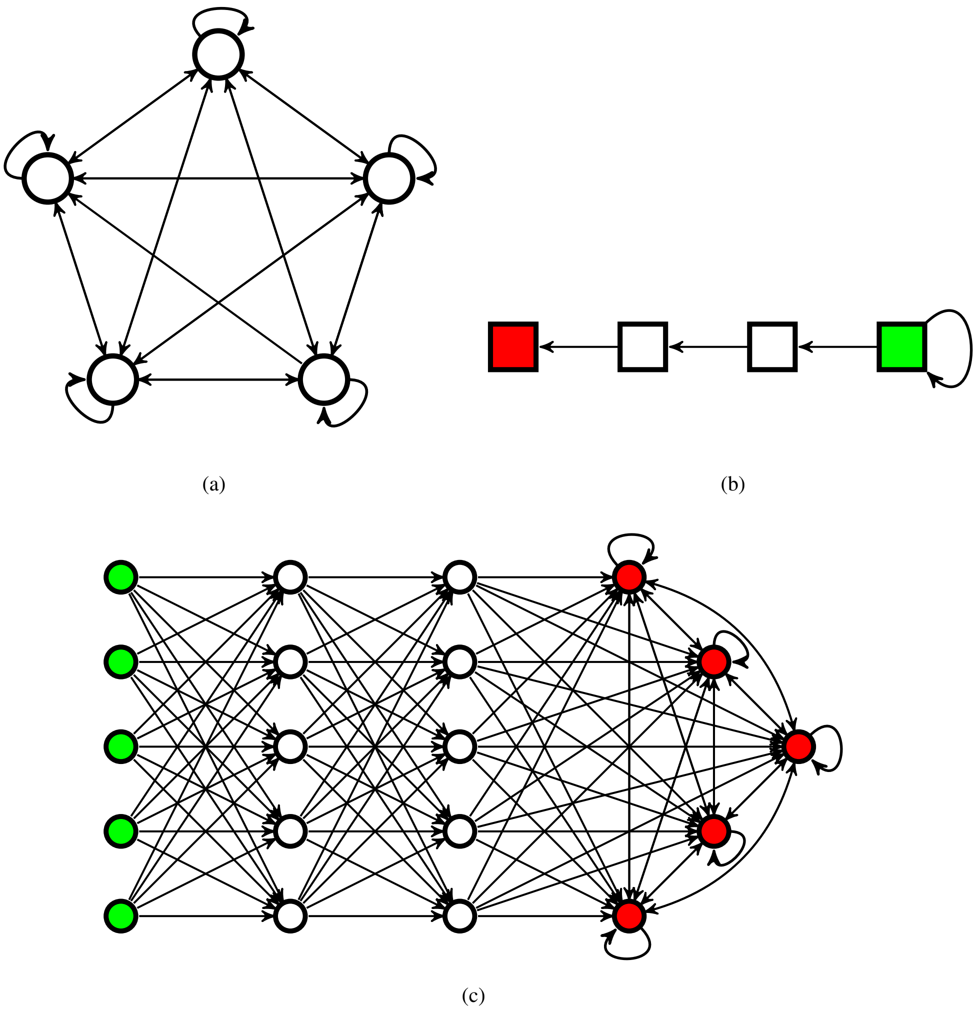

Figure 2. An additional example of a belief system. A product of a complete graph/cycle graph with 5 nodes and a path graph of 4 logical belief constraints. (a) A complete graph with 5 agents. (b) A directed path graph with 5 nodes. (c) The belief system graph from the network of agents in (a) and the network of logic constraints in (b).

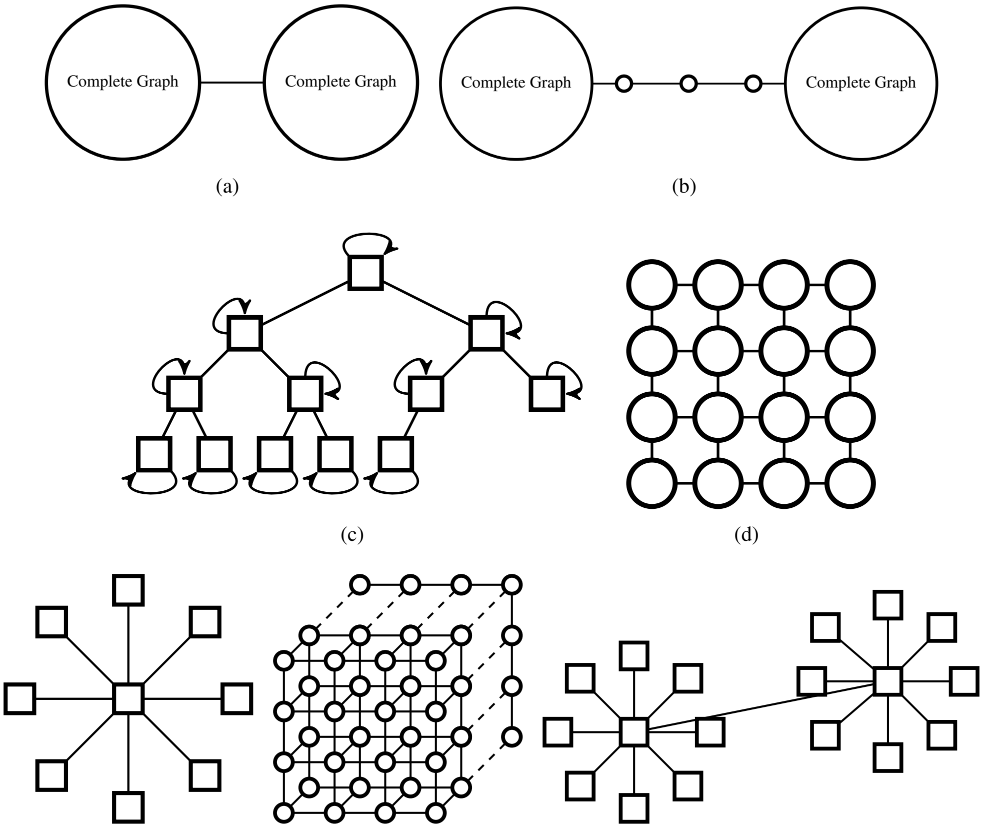

(a) (b) (c) (d) (e) (f) (g)

Figure 3. Examples of common graph families. (a) Dumbbell graph, two complete graphs connected by an edge. (b) Bolas graph, two complete graphs connected by a path. (c) Complete binary tree. (d) 2-d grid or lattice. (e) Star graph. (f) 3-d grid. (g) Two-star graph connected to their centers.

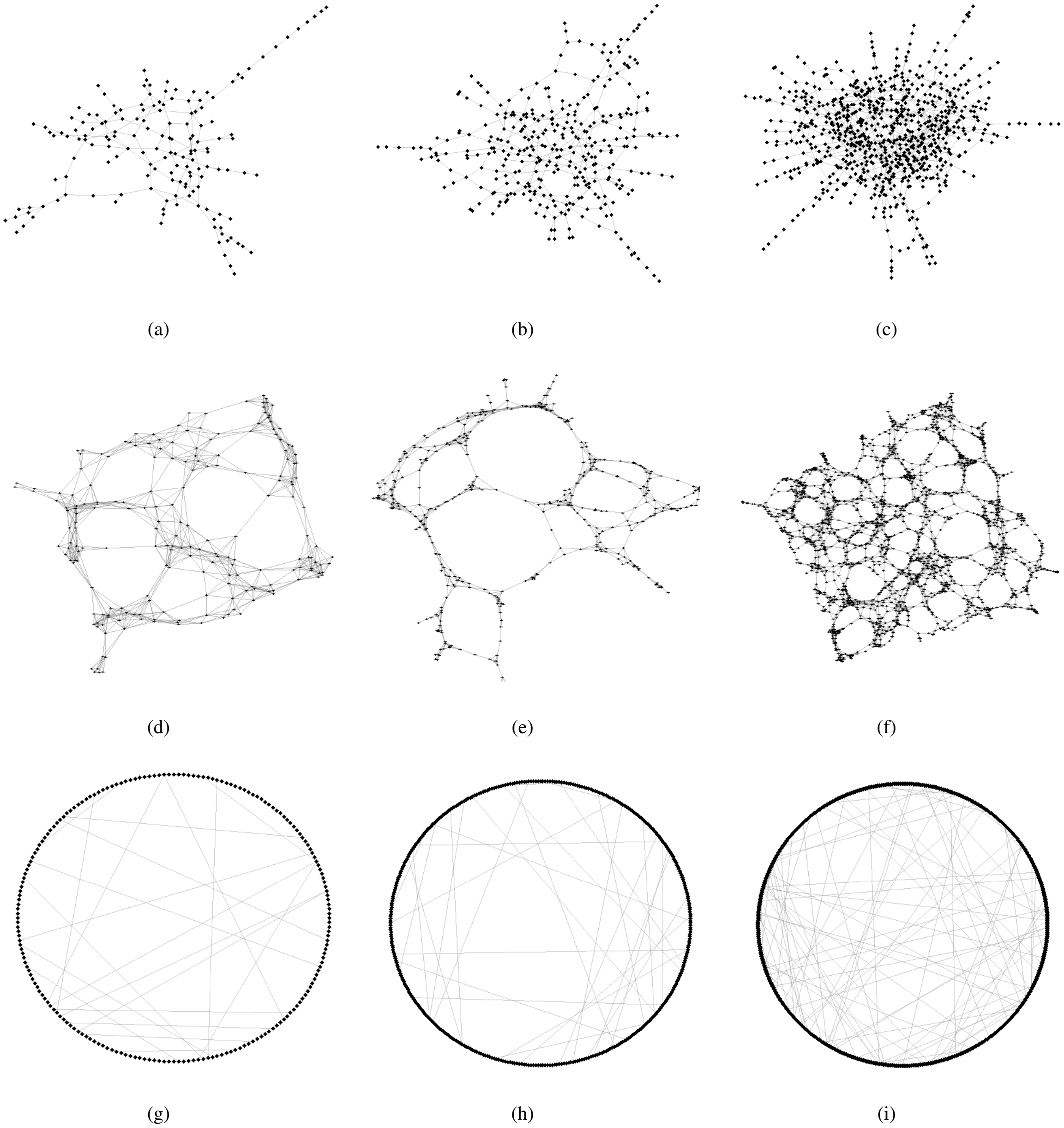

Figure 4. Examples of random graphs. (a-c) Geometric random graphs with 200, 400 and 2000 nodes respectively. A geometric random graph is a result of randomly placing n nodes in a metric space and adding an edge between two nodes if and only if their distance is smaller than certain radius r[7]. (d-f) Erdős-Rényi random graphs with 200, 400 and 1000 nodes respectively. An $\mathscr{G}_{n,p}$ Erdős-Rényi graph is the result of adding edges independently with probability p to a set of n nodes[8]. (g-i) Newman-Watts Random Graphs with 200, 400 and 1000 nodes respectively. The Newman-Watts graph $H_{n,k,p}$ is the random graph obtained from a (n,k)-ring graph by independently adding edges with probability p[9].

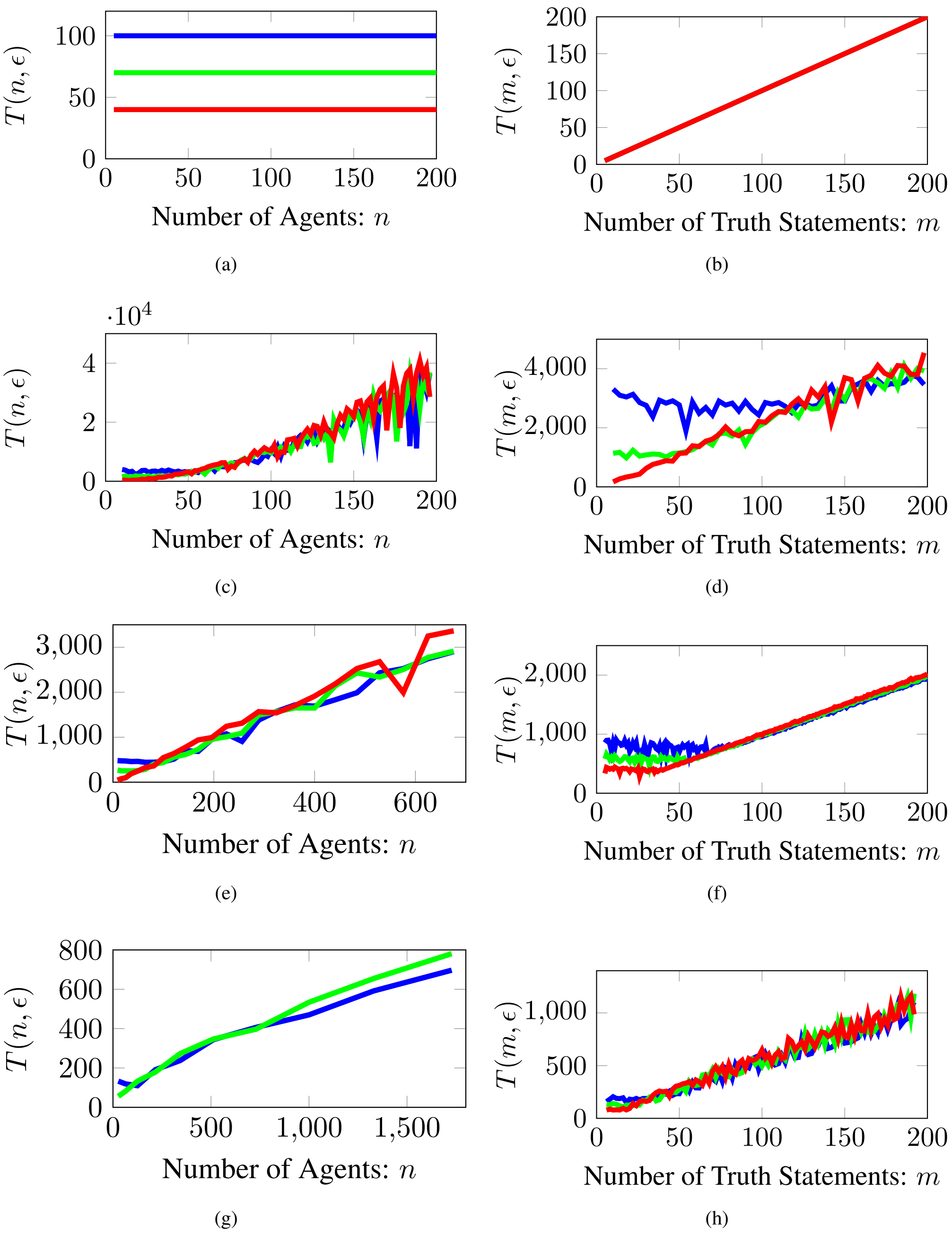

Figure 5. Convergence time for different examples of networks of agents and network of truth statements in a belief system. Varying the number of agents for a: (a) complete graph, (c) dumbbell graph, (e) 2-d grid and (g) 3-d grid. Varying the number of truth statements for a: (b) directed path, (d) complete binary tree, (f) star graph and (h) two joined star graphs.

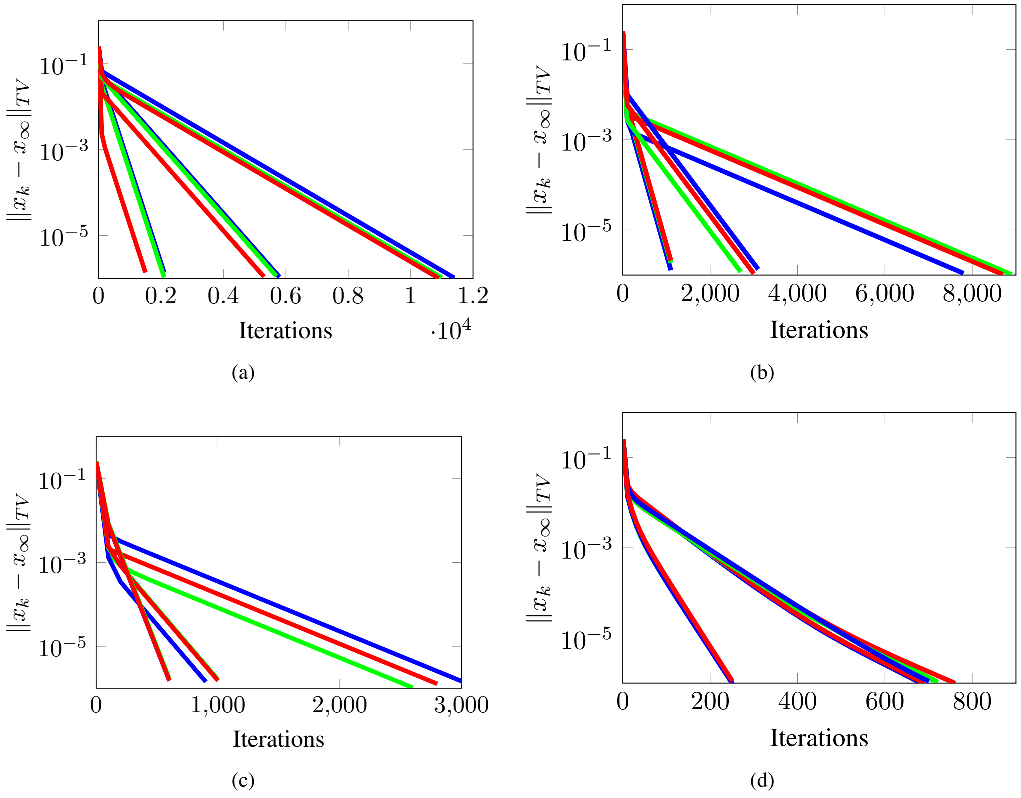

Figure 6. Linear convergence of the belief system. Distance to the final value of a belief system with: (a) a directed cycle network of agents and a directed path of truth statements, (b) a dumbbell network of agents and a complete binary tree of truth statements, (c) a 2-d grid of agents and a star network of truth statements, (d) a 3-d grid of agents and a two-jointed star network of truth statements.

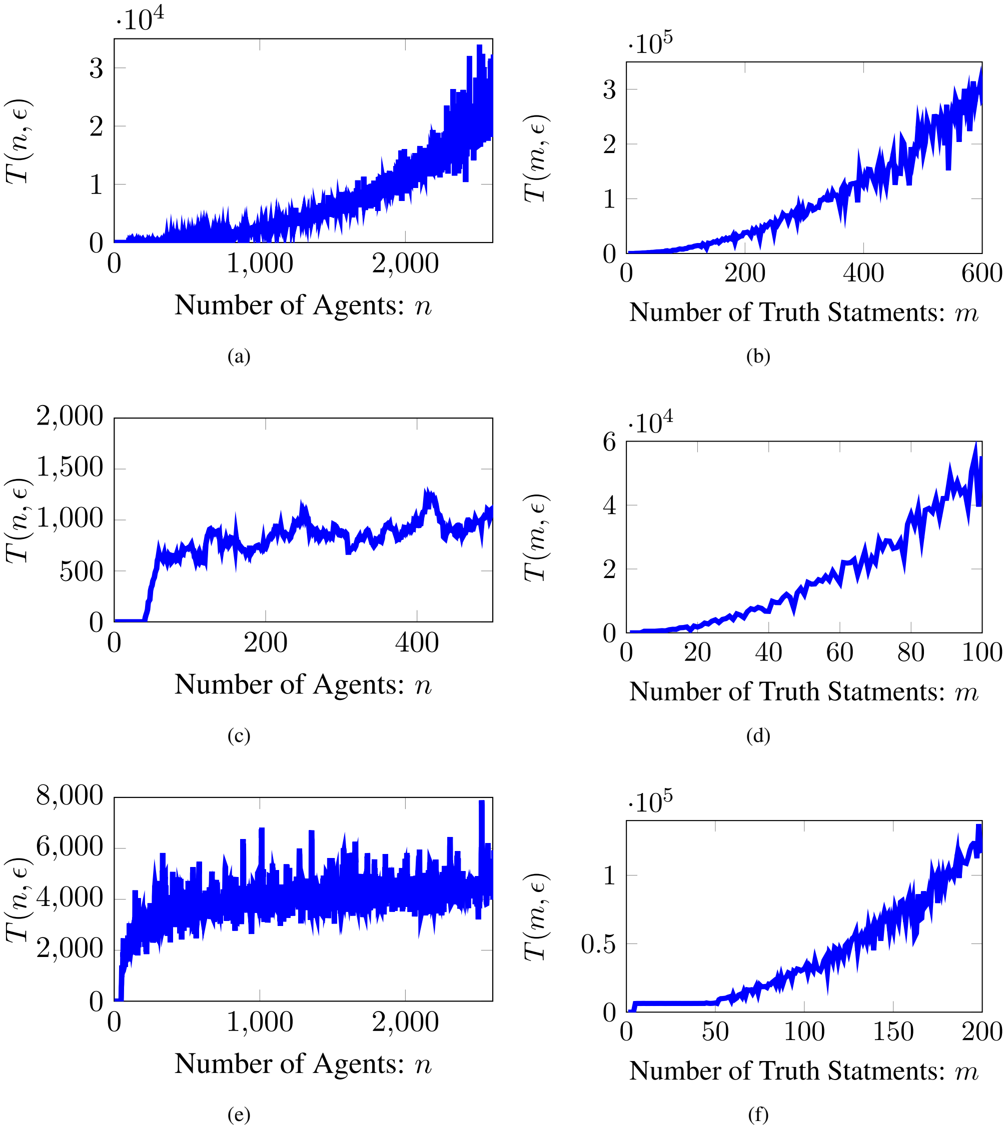

Figure 7. Convergence time dependency for random graphs. (a) Varying the number of agents in a geometric random graph with a fixed number of truth statements in a Bolas graph. (b) Varying the number of truth statements in a Bolas graph with a fixed number of agents in a geometric random graph. (c) Varying the number of agents in an Erdős-Rényi random graph with a fixed number of truth statements in a dumbbell graph. (d) Varying the number of truth statements in a dumbbell graph with a fixed number of agents in an Erdős-Rényi random graph. (e) Varying the number of agents in a Newman-Watts random graph with a fixed number of truth statements in an undirected path graph. (f) Varying the number of truth statements in an undirected path graph with a fixed number of agents in a Newman-Watts random graph random graph.

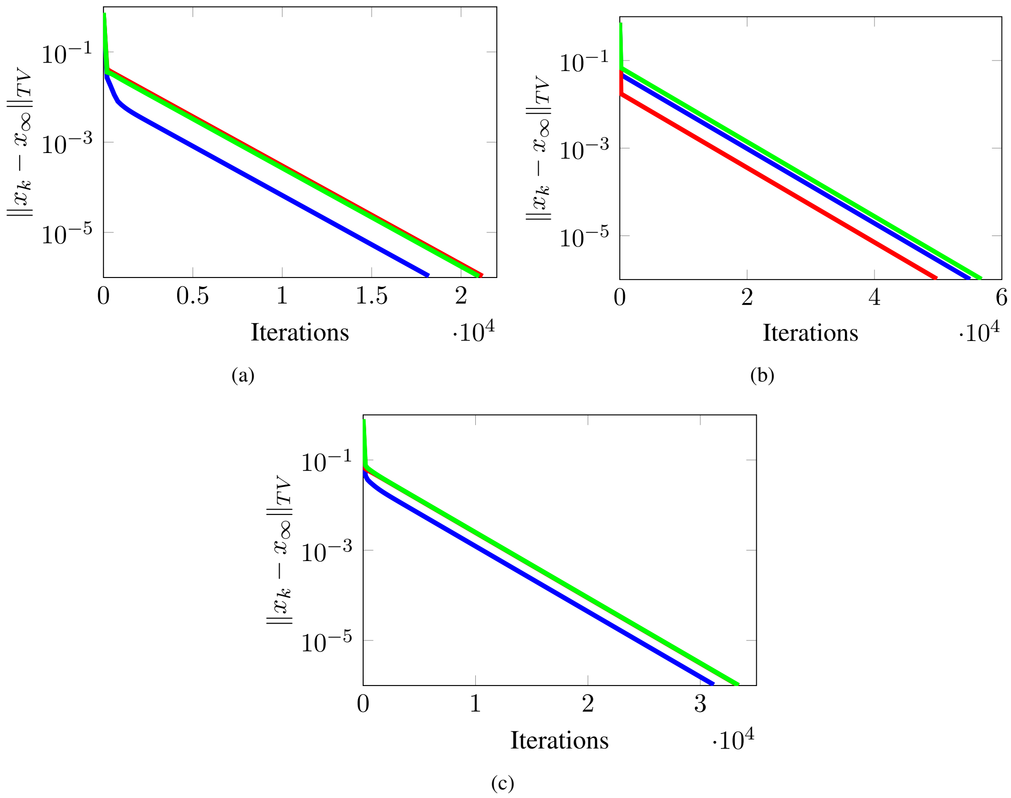

Figure 8. Linear convergence rate of the belief system with random networks of agents. (a) Distance to the stationary distribution for a network of 200 agents modeled as a geometric random graph and a network of 150 truth statements modeled as a Bolas graph. (b) Distance to the stationary distribution for a network of 500 agents modeled as an Erdős-Rényi random graph and a network of 100 truth statements modeled as a dumbbell graph. (c) Distance to the stationary distribution for a network of 500 agents modeled as a small-world random graph and a network of 100 truth statements modeled as an undirected path graph.

Table 1. Upper bounds on the mixing time for various graph topologies.

Network Topology	Mixing Time
Cycle [6] Section 5.3.1	$O(n^2)$
Path [10,11]	$O(n^2)$
Star Graph [11]	$O(1)$
Two Joined Star Graphs	$O(1)$
Dumbbell Graph [12]	$O(n^2)$
Lollipop [13]	$O(n^2)$
Bolas Graph [13]	$O(n^3)$
Complete Binary Tree [6] Section 5.3.4	$O(n)$
k-d Hypercube $\{0,1\}^k$ [6] Section 5.3.3	$O(k \log k + k)$
L-Lattice on $\mathscr{Z}_n \times \mathscr{Z}_n$ [14,15]	$O(n^2)$
2-d Grid [14,15]	$O(n(\log n+))$
3-d Grid [14,15]	$O(n^{2/3}(\log n+))$
k-d Grid [14,15]	$O(2k^2 n^{2/k}(\log n+))$
2-d Torus [6] Section 5.3.3	$O(n^2)$
3-d Torus [6] Section 5.3.3	$O(n^2)$
k-d Torus [6] Section 5.3.3	$O(k^2 n^2)$
Eulerian Graph [16]	$O(\|E\|^2)$
Lazy Eulerian with degree d-degree [17]	$O(n\|E\|)$
Eulerian: d-degree, max-degree weights and expansion [16]	$O(n^2 d)$
Geometric Random Graph: $\mathscr{G}^d(n,r)$ [18]	$O(r^{-2} \log n)$
Geometric Random Graph: $\mathscr{G}^2(n, \Omega(\text{polylog}(n)))$ [19]	$O(\text{polylog}(n))$
Erdős-Rényi: $\mathscr{G}(n, c/n)$, $c > 1$ [20,21]	$O(\log^2 n)$
Erdős-Rényi: $\mathscr{G}(n, (1+\delta)/n)$, $\delta^3 n \to \infty$ [22,23]	$O((1/\delta^3) \log^2(\delta^3 n))$
Erdős-Rényi: $\mathscr{G}(n, 1/n)$ [24]	$O(n)$
Newman-Watts (small-world) Graph [25]	$O(\log^2 n)$
Expander Graph [26]	$O(\log n)$
Any Connected Undirected Graph with Metropolis weights [27]	$O(n^2)$
Any Connected Graph	$O(\|E\| \text{diam}(\mathscr{G}))$

12. Kannan, R., Lovász, L. & Montenegro, R. Blocking conductance and mixing in random walks. *Comb. Probab. Comput.* **15**, 541–570 (2006).

13. Aldous, D. & Fill, J. Reversible Markov chains and random walks on graphs (2002).

14. Avin, C. & Ercal, G. Bounds on the mixing time and partial cover of ad-hoc and sensor networks. In *EWSN*, 1–12 (2005).

15. Chandra, A. K., Raghavan, P., Ruzzo, W. L., Smolensky, R. & Tiwari, P. The electrical resistance of a graph captures its commute and cover times. *Comput. Complex.* **6**, 312–340 (1996).

16. Montenegro, R. The simple random walk and max-degree walk on a directed graph. *Random Struct. & Algorithms* **34**, 395–407 (2009).

17. Boczkowski, L., Peres, Y. & Sousi, P. Sensitivity of mixing times in Eulerian digraphs. *arXiv preprint arXiv:1603.05639* (2016).

18. Boyd, S. P., Ghosh, A., Prabhakar, B. & Shah, D. Mixing times for random walks on geometric random graphs. In *ALENEX/ANALCO*, 240–249 (2005).

19. Avin, C. & Ercal, G. On the cover time and mixing time of random geometric graphs. *Theor. Comput. Sci.* **380**, 2–22 (2007).

20. Benjamini, I., Kozma, G. & Wormald, N. The mixing time of the giant component of a random graph. *Random Struct. & Algorithms* **45**, 383–407 (2014).

21. Fountoulakis, N. & Reed, B. The evolution of the mixing rate. *arXiv preprint math/0701474* (2007).

22. Ding, J., Kim, J. H., Lubetzky, E. & Peres, Y. Anatomy of a young giant component in the random graph. *Random Struct. & Algorithms* **39**, 139–178 (2011).

23. Ding, J., Lubetzky, E., Peres, Y. *et al.* Mixing time of near-critical random graphs. *The Annals Probab.* **40**, 979–1008 (2012).

24. Nachmias, A. & Peres, Y. Critical random graphs: diameter and mixing time. *The Annals Probab.* 1267–1286 (2008).

25. Addario-Berry, L. & Lei, T. The mixing time of the Newman-Watts small-world model. *Adv. Appl. Probab.* **47**, 37–56 (2015).

26. Durrett, R. *Random Graph Dynamics* (Cambridge University Press, UK, 2007).

27. Olshevsky, A. Linear time average consensus and distributed optimization on fixed graphs. *SIAM J. on Control. Optim.* **55**, 3990–4014 (2017).
